# Social mixing patterns of United States healthcare personnel at a quaternary health center: a prospective observational study

**DOI:** 10.1017/ice.2024.234

**Published:** 2025-01-30

**Authors:** Lauren Pischel, Obianuju G Aguolu, Noureen Ahmed, Melissa M Campbell, Ryan Borg, Chelsea Duckwall, Kathryn Willebrand, Agnieska Zaleski, Elliott E Paintsil, M. Catherine Muenker, Amyn A Malik, Moses C Kiti, Joshua L Warren, Samuel M Jenness, Ben A Lopman, Justin Belsky, Richard A Martinello, Inci Yildirim, Albert I Ko, Saad B Omer

**Affiliations:** 1 Department of Internal Medicine, Section of Infectious Diseases, Yale School of Medicine, New Haven, CT, USA; 2 Yale Institute for Global Health, New Haven, CT, USA; 3 Department of Epidemiology, Ohio State University College of Public Health, Columbus, OH, USA; 4 University of Texas Southwestern Medical Center, O’Donnell School of Public Health, Dallas, TX, USA; 5 Department of Pediatrics, Duke University Hospital, Durham, NC, USA; 6 Department of Epidemiology of Microbial Diseases, Yale University School of Public Health, New Haven, CT, USA; 7 Yale Center for Clinical Investigation, New Haven, CT, USA; 8 Medical College of Wisconsin, Milwaukee, WI, USA; 9 Rollins School of Public Health, Emory University, Decatur, GA, USA; 10 Department of Biostatistics, Yale University School of Public Health, New Haven, CT, USA; 11 Department of Emergency Medicine, Yale School of Medicine, New Haven, CT, USA; 12 Department of Pediatrics, Pediatric Infectious Diseases, Yale School of Medicine, New Haven, CT, USA

## Abstract

**Background:**

Understanding healthcare personnel’s (HCP) contact patterns are important to mitigate healthcare-associated infectious disease transmission. Little is known about how HCP contact patterns change over time or during outbreaks such as the COVID-19 pandemic.

**Methods:**

This study in a large United States healthcare system examined the social contact patterns of HCP via standardized social contact diaries. HCP were enrolled from October 2020 to June 2022. Participants completed monthly surveys of social contacts during a representative working day. In June 2022, participants completed a 2-day individual-level contact diary. Regression models estimated the association between contact rates and job type. We generated age-stratified contact matrices.

**Results:**

Three-hundred and sixty HCP enrolled, 157 completed one or more monthly contact diaries and 88 completed the intensive 2-day diary. In the monthly contact diaries, the median daily contacts were 15 (interquartile range (IQR) 8–20), this increased slightly during the study (slope-estimate 0.004, p-value 0.016). For individual-level contact diaries, 88 HCP reported 2,550 contacts over 2 days. HCP were 2.8 times more likely to contact other HCP (n = 1,592 contacts) than patients (n = 570 contacts). Rehabilitation/transport staff, diagnostic imaging technologists, doctors, nurses, mid-level, and laboratory personnel had higher contacts compared with the lowest contact group (Nursing aids). Contact matrices concentrated in working-age populations.

**Conclusions:**

HCP contacts concentrate in their work environment, primarily with other HCP. Their contacts remained stable over time even during large changes to societal contact patterns during the COVID-19 pandemic. This stability is critical for designing outbreak and pandemic responses.

## Introduction

Healthcare settings are unique and high-risk environments for infectious disease transmission. These settings concentrate diverse groups of individuals including susceptible and often immunocompromised patients with healthcare personnel (HCP) in high-contact density (eg high number of interconnected contacts) and high-contact intensity (eg long duration or close physical contacts) environments. These factors all contribute to potential healthcare-associated infections (HAIs) including from drug resistant or even high-consequence pathogens.^1^

HAIs are regrettably common and are estimated to affect 3.2% of hospitalized patients.^2,3^ Multiple infectious agents spanning from high-consequence pathogens such as Ebola viruses, Marburg virus, SARS-CoV-1 to more routine pathogens such as SARS-CoV-2, Norovirus, and Clostridioides difficile have the potential for transmission within healthcare settings.^4-7^ These infections are costly to healthcare systems, costing the United States an estimated $36 - $45 billion dollars annually, though this figure is likely as underestimate as it does not include healthcare-associated respiratory pathogen infections. Critically, HAIs result in unanticipated morbidity and mortality for patients.^8,9^ Healthcare-associated COVID-19 infection is associated with increased risk of intensive care unit admission and increased time to discharge in the post-Omicron era.^10^

In outbreaks with novel pathogens, HCP bear the brunt of infections; up to 40% of the initial cases of MERS and SARS-CoV-1 were in HCP, and 15% of SARS-CoV-2 cases.^11,12^ When large numbers of HCP fall ill, this causes great strain on already thinly stretched healthcare services with potentially catastrophic results.^13,14^

HCP can initiate or propagate hospital transmission chains to patients and staff.^15-17^ For SARS-CoV-2, HCP can be asymptomatic, pauci-symptomatic or pre-symptomatic hosts who transmit SARS-CoV-2 to susceptible or immunosuppressed patients.^18^ Transmission between HCP is another form of HAI, often during close-contact such as un-masked encounters such as in break-rooms.^19^ Additionally, HCP can initiate community transmission chains.^20^

Gaining a superior understanding of HCP contact patterns can help develop informative infectious disease transmission models in the healthcare environment. Classic compartmental infectious disease models operate under the assumption of homogenous mixing, an assumption that is violated in the healthcare environment.^21,22^ Hospitals have dense and strongly clustered contact networks with HCP as important nodes of contact with greater number and greater intensity of contacts compared to other personnel.^23,24^ This can inflate the effective reproduction number.^25^ Prior studies using either self-reported contact diaries or wearable sensors showed HCP have nonrandom mixing at work, and this mixing depends largely on their job description.^22,25-27^ How contact patterns of HCP in the United States changed during and after the COVID-19 pandemic, and detailed descriptions of contacts by job description are not known.

Our aim was to characterize the social contact patterns of HCP by demographics, job, and work location in a quaternary healthcare system in the United States using standardized social contact diaries. Enrollment opened October 2020. We collected monthly contact diaries from January 2021 to May 2022 with an intensive individual-level 2-day contact diary in June 2022. Here we report the longitudinal data and intensive diary contact patterns.

## Methods

### Study site and participants

This longitudinal observational study took place from October 20, 2020, to June 1, 2022, at a large healthcare system in the United States with a focus on the central quaternary care hospital which also provided general hospital services to the surrounding communities (Supplemental Figure 1, Supplementary Table 1). Individuals 18 years of age or older and employed through or working in the health system were eligible to participate. Individuals who were not actively working (eg on leave) during the study period were excluded. Participants were recruited from an existing cohort consisting of volunteer HCP who had serial monitoring for SARS-CoV-2 infection.^28^ Additional participants were recruited at targeted in-person events, flyers, virtual townhalls, and email. Surveys were completed via Qualtrics.^29^ Study participants were provided gift card incentives from $40 to $100 depending on the degree of participation.

Participants were enrolled from October 20, 2020, to June 1, 2022 (Supplemental Table 2). Starting January 2021, participants reported monthly contact data (Supplemental Table 3). From May 30 to June 5, 2022 (the “June contact diary”) participants completed an intensive individual-level 2-day (48-hour) contact diary (Supplemental Table 4). Participants’ jobs were summarized into 12 categories (Supplemental Table 2). The enrollment goal was 250 individuals.

### Contact diaries

Participants completed longitudinal contact diaries each month reporting on one representative working day (24-hour period) of the participants choosing. The total number of contacts per day was summarized as follows: direct contacts with patients per day, number of indirect contacts with patients per day, the average number of minutes spent with a patient on that day, number of direct contacts with HCP per day, and how the number of contacts this week compared to a typical week. The survey documented participant demographics, work location(s), COVID-19 related symptoms in the past month, duration of illness, working with symptoms shifts on a COVID-19 unit, and other COVID-19 exposure to patients. In the June contact diary direct contacts were further divided into physical contact, non-physical contact, and direct proximity and individual-level information about the contact (contact duration and contact demographics) was also collected. If the participant contacted the same individual over 2 days, this was recorded as 2 separate contacts.

### Definitions

Direct contact was defined as contact within 6 feet of another individual for at least 2 minutes. Two minutes was chosen as the lower limit of time needed to transmit viruses via aerosols in a closed indoor environment.^30^ We defined indirect contact as being in the same room but further than 6 feet away. Physical contact was defined as directly touching someone or their clothes. Nonphysical contact was a two-way conversation with three or more words exchanged in the physical presence of another person (within approximately 6 feet of each other), but with no physical contact. Direct proximity was defined as being within 6 feet of the contact for 20 seconds or more with neither conversation nor physical contact. Twenty seconds was chosen to capture some of the quickest social interactions and 2 meters/ 6 feet as this is the estimated proximity for transmission for infectious respiratory particles.^31-33^

### Statistical methods

Enrollment forms were excluded at < 30% completion. Demographics of participants were assessed monthly for differential loss to follow-up. The total number of contacts over time was assessed with negative binomial regression with the null hypothesis of no change over time (eg slope = 0, statistical significance level (ie α) of 0.05). The association between total number of contacts and participant’s job was assessed via negative binomial regression that included calendar month to capture temporal trends via cubic splines and individual-specific HCP random effects. A mixed-effects ordinal logistic regression was performed with a similar structure for the total estimated direct contacts with patients, total indirect contacts with patients, total time with patients, and total direct contacts with HCP.

From the individual-level June 2022 contact diaries, contact relationship, contact type, location, and duration were compared to participant job via the Kruskal–Wallis test. If the null hypothesis of no-difference of contacts by job was rejected (α = 0.05), a post hoc comparison was done with the Dunns test using the Bonferroni method of controlling for multiple comparisons. A Wilcoxon signed-rank test compared the total number of contacts in detail vs the total summative contacts.

Contact matrices were generated for the average number of contacts per day by decade of participant age vs estimated decade of contact age. Contact matrices were assessed for age-assortativeness (eg how often HCW and their contact ages were within the same estimated decade) by comparing the mean and standard deviation (SD) of proportion of contacts in the same age range as the HCP participant.^34^ This study is reported according to STROBE guidelines (Supplemental Table 5) and approved by the Yale University Institutional Review Board (#2000028924).^35^ To protect participant privacy, summative data is available at https://github.com/lpischel/hcpmix and contact matrices at https://socialcontactdata.org/data or https://zenodo.org/records/14156576 Full data available upon request with appropraite data use agreements. All analyses were conducted in R V.4.2.1.^36^

## Results

Overall, 360 HCP completed the enrollment questionnaire, 168 (47%) completed the enrollment survey, 95 (26%) completed longitudinal contact diaries alone, 35 (9.7%) completed the June contact diary that contained individual-level contact information, and 62 (17%) individuals completed both longitudinal and the June contact diary. Of the 97 intensive 2-day individual-level contact diaries, 88 completed the survey with both summative and individual-level contacts (Table 1; Supplemental Table 6). The median age of participants was 38 years (interquartile range (IQR) 30–49 yr), and 304 (84%) were women. The most common profession was nurse (134, 37%). The median follow-up time was 20.5 days (IQR 13–124 days).

### Longitudinal contact diaries

In total, 519 longitudinal contact diaries were started by 195 HCP, 45 diaries did not provide any data and were excluded. Six were excluded as they were completed after the study closed. Of the remaining 469 diaries from 181 HCP, 417 (89%) were complete (Supplemental Table 7). Participation declined over time, with a large increase prior to June 2022 with additional recruitment efforts. For the longitudinal summative contact diaries, the median number of diaries completed per HCP was 2 (IQR 1–3, maximum 15).

**Figure 1.**
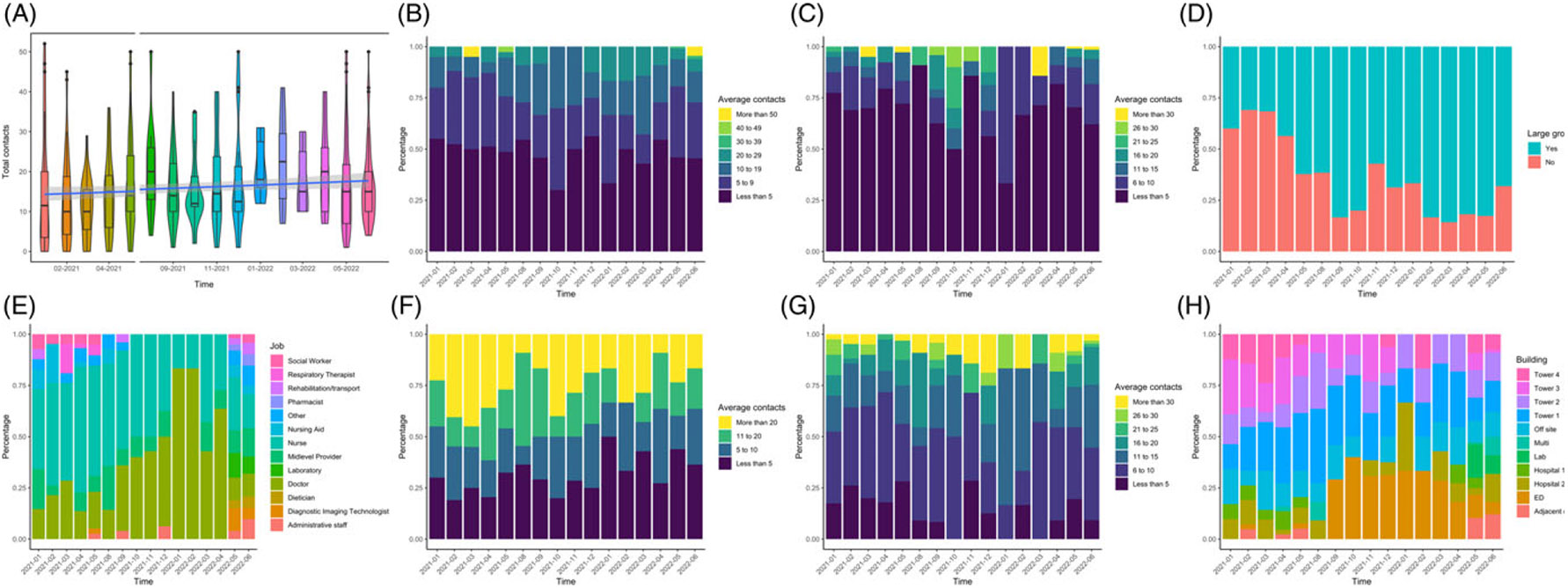
Results of summative contacts of health care workers are shown from January 2021 to June 2022. Contacts were reported over a representative working day (24-hour period) as chosen by the Health Care Personnel (HCP). As the study was closed in June and July 2021, these months are excluded. HCP provided summative information about the number and type of contacts over time. **A:** Number contacts per day over time shown in violin and box plots. Negative binomial regression shown in blue line with 95% CI in gray. **B:** The average number of direct contacts a HCP had with patients per day is shown as a percent stacked barplot where month is on the x-axis and percentage of responses on the y-axis. **C:** Average number of indirect contacts a HCP had with patients per day. **D:** If the HCP attend a large group gathering where individuals could not be individually identified during the reported day. **E:** Job of HCP on reported contact diary over time. **F:** Average contact time HCP spent with an average patient per day over time. **G:** Average number of direct contacts a HCP had with other HCP per day. **H:** Building worked by HCP on the reported day in over time.

The median contacts per day were 15 (IQR 8–20, unknown = 23), this did increase significantly over the course of the study but not by a large absolute amount (increase in 0.0004 contacts per month, *P* = 0.016) as assessed by negative binomial regression (Figure 1). Nursing to patient ratios remained stable over the course of the study. Nearly half of respondents had direct contact with less than 5 patients. Indirect contact with patients was reported less frequently with 70% (n = 313) of participants recording indirect contact with 5 or less patients. Seventeen percent of HCP (n = 74, unknown = 28) reported direct contacts with less than 5 other HCP, and 38%, 20% 12%, and 13.1% for contact with 6–10, 11–15, 16–20, and 21 or more other HCP, respectively (Supplemental Table 8).

### Regressions

Rehabilitation/transport staff, diagnostic imaging technologists, doctors, nurses, mid-level, and laboratory personnel had greater numbers of total contacts compared with the lowest contact group (Nursing aids) (Table 2). The number of direct patient contacts and time spent with patients, compared with laboratory personnel, all jobs had greater direct contacts with patients except for those working in administrative support. For indirect patient contacts, there was an inadequate distribution of responses for an ordinal regression. There was no significant difference of contact with other HCP by job.

### Individual-level contact diaries

**Figure 2.**
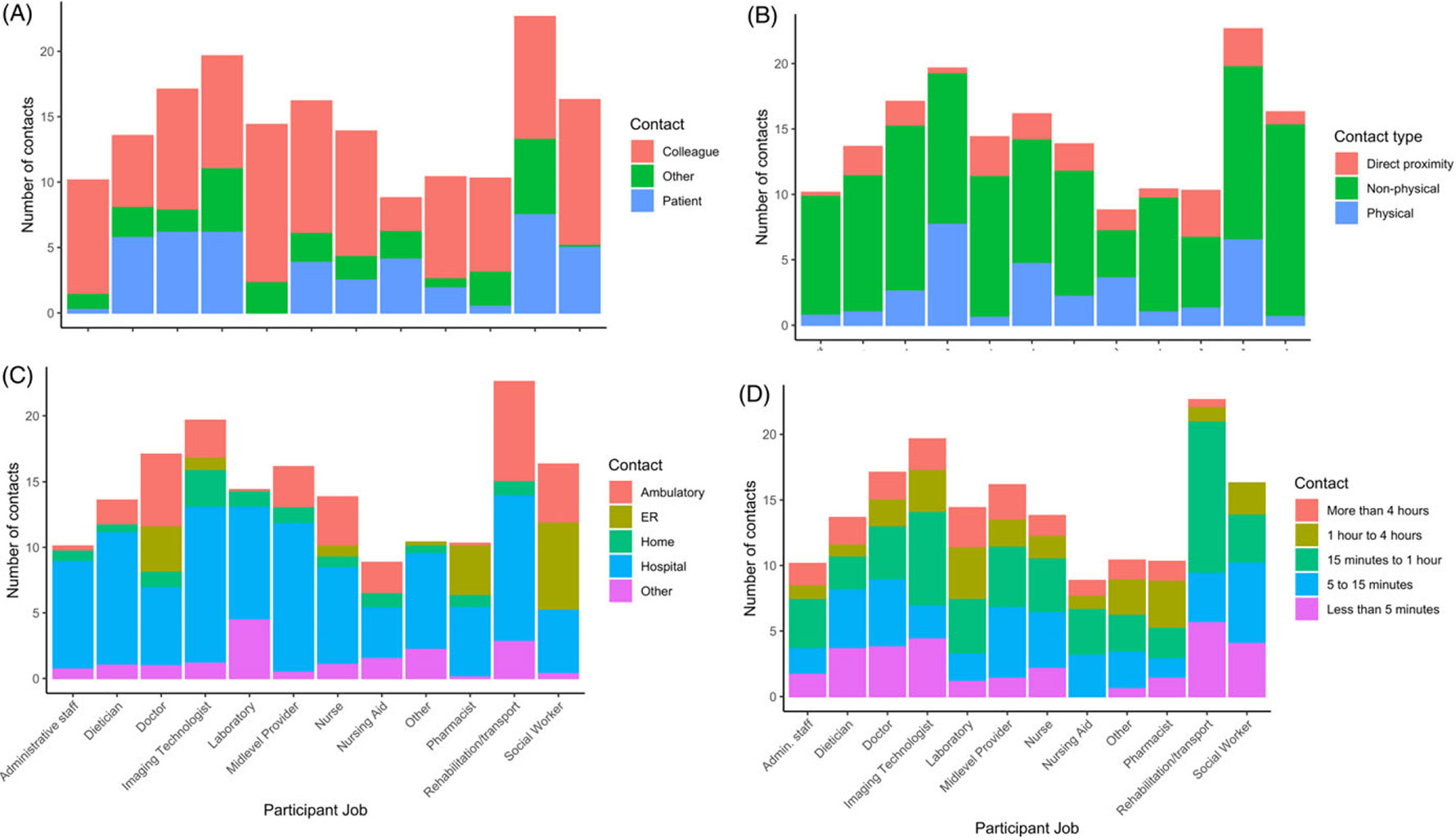
Individual reported contacts per day of HCP are summarized from the 2-day intensive contact diary as stacked barplots in June 2022. **A.** Number of contacts per HCP per day by participant job and relationship to contact individual. **B:** Number of contacts per HCP per day by participant job and contact type (direct-proximity, non-physical, or physical contact). **C:** Number of contacts per HCP per day by participant job and location of the contact. **D:** Number of contacts per HCP per day by participant job and duration of the contact.

For individual-level contact diaries in June 2022, 2,550 contacts were reported over 2 days (Supplemental Table 9). The median number of contacts reported per HCP per day was 12.75 IQR [8.4 – 18.8]. Most contacts were with other colleagues (1,592, 62.4%). Rehabilitation and transport staff had the highest contact number with patients (7.8, IQR 4.9–10.4), while social workers had the highest contact per day with other HCP (12, IQR 10.3–12.5) (Figure 2). Rehabilitation and transport personnel also reported the highest number of non-physical contacts (14.8, IQR 7.6–20.4) and physical contacts (12.5, IQR 6.8–12.5). Direct proximity and non-physical contact did not vary by profession. For physical contacts, diagnostic imaging technologists had higher physical contacts than laboratory personnel (p-value < 0.05). Rehabilitation or transport personnel the highest number of contacts between 15 minutes and 1 hour (14.3, IQR 10.3–15.5).

### Contact matrices

**Figure 3.**
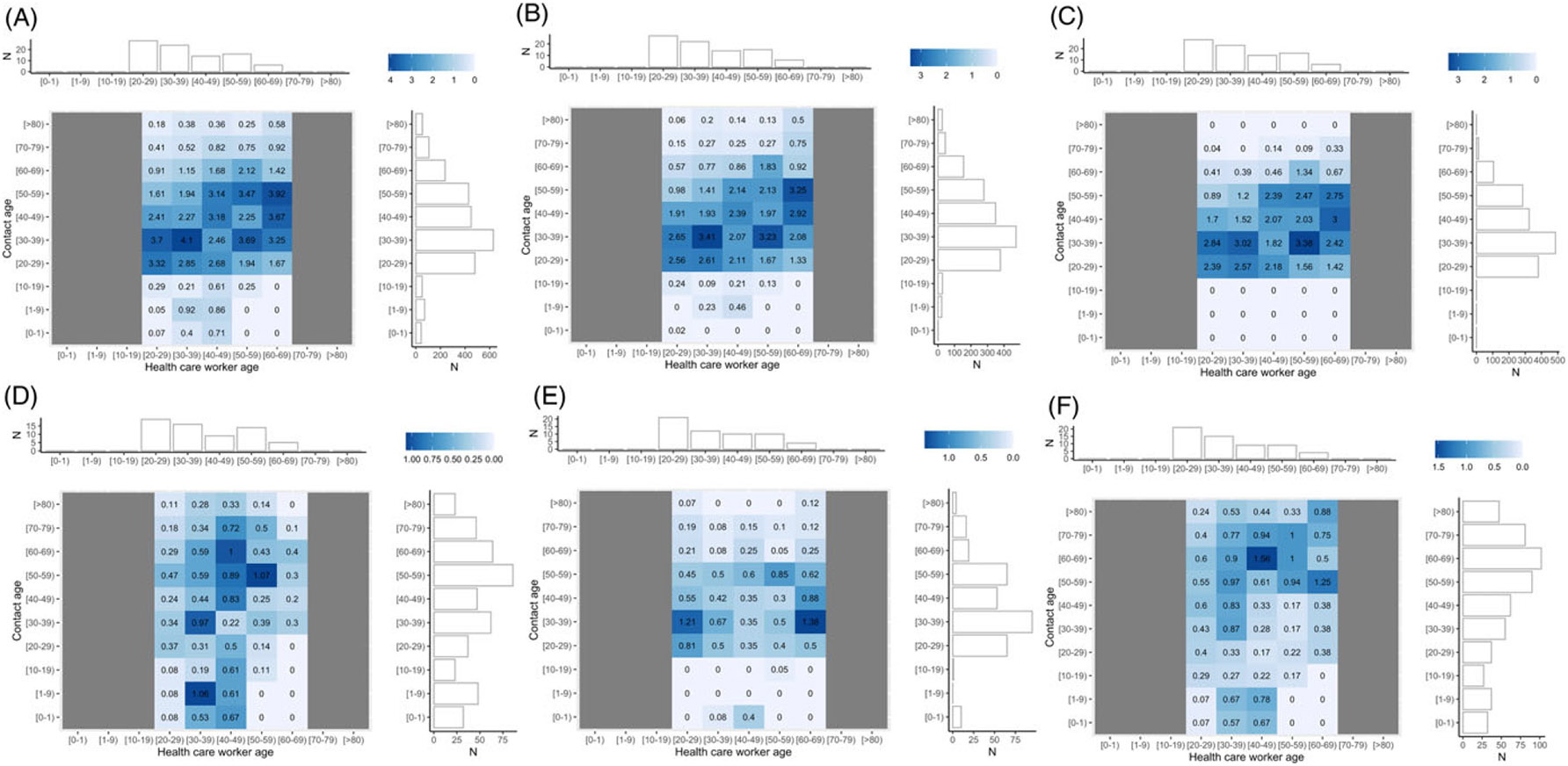
The x-axis shows the HCP age with non-working ages grayed out (eg less than 20, greater than 70), and the y-axis shows the estimated contact age. Each tile is filled and colored with the average number of contacts per HCP of that age range with contacts of that age range per day. A horizontal marginal histogram of each contact matrix shows the age distribution of HCP and a vertical marginal histogram shows the age distribution of contacts. Please note each plot has a different scale, this is to preserve the ability to discriminate different values. **A:** Average contacts of HCP with all individuals per day (n = 2,550). **B:** Average non-physical contacts per day (n = 1,754). **C:** Average contacts per HCP with other HCP per day (n = 1,592). **D:** Average physical contacts per day (n = 462). **E:** Average direct proximity contacts per day (n = 328). **F:** Average contacts per HCP with patients per day (n = 570).

Contacts for HCP were spread across all age ranges (Figure 3). Physical contacts and contacts with patients focused on younger and older ages. Contacts with other HCP were distributed across all working ages regardless of contact type (Supplemental Figure 3). The age-contact matrix of all contacts was not age-assortative: the average within age-group proportion of contact was 21.2% (SD 7.4%).

### Sensitivity analysis

In the June 2022 diary, the total number of summative contacts was compared with the detailed number of daily contacts: in 76.6% of cases this was the same value. In those that differed, 58.5% had more contacts reported in detail than in summative reporting. By paired Wilcoxon signed-rank test, the null hypothesis that the number of contacts reported in detail and in sum are the same was not rejected (p-value 0.7) (Supplemental Figure 4).

## Discussion

This prospective observational study quantified social contacts of HCP at a large quaternary health center with monthly summative contact diaries and a 2-day individual contact diary in June 2022. Our findings highlight that HCP’s contacts remained relatively stable during the COVID-19 pandemic response after January 2021. Though contacts between HCP and patients are often considered the highest risk for pathogen transmission, HCP were 2.8 times more likely to contact another HCP as opposed to patients. In the individual-level contact diary in June 2022, the type and number of contacts varied by job. Rehabilitation and transport personnel had the highest number of both nonphysical and physical contacts. Social workers had the greatest number of contacts with their colleagues while rehabilitation and transport personnel had the greatest contact with patients. HCP contacts with other HCP concentrated in working age ranges while contacts with patients focused on the extremes of age which mirrors the ages of most patients.

Contact patterns of HCP are difficult to characterize due to the complex ecosystem of contact within the healthcare system. Other studies have reported that either nurses or doctors have the greatest frequency of direct contacts with patients but have not reported in detail contact patterns of other professions in the hospital.^22,23,37,38^ Prior studies have used electronic medical record (EMR) data to reports that residents and nurses had the highest degree of contacts with other HCP.^37^ Shirreff et al. used sensors in France to simulate SARS-CoV-2 outbreaks across different hospital wards from April to June 2020 and reported that targeting interventions toward those with the highest cumulative contact hours with patients most effectively limited HAI transmission.^39^ Wilson-Aggarwal et al. using EMR data and door-logs in a London academic hospital reported a drop in total patient contacts during the first wave of COVID-19 (March to June 2022); however, the rate of patient contact remained stable throughout the pandemic.^38^ Our results show similar stability, though our contact measurements began after the first wave of COVID-19. Building on prior work, we were able to describe with greater detail the variation of contact patterns by 12 occupations and highlight the importance of high contact individuals whose jobs span multiple units in the hospital.

Though greater risk may be perceived through patient contact, HCP have more frequent contact with fellow HCP, resulting in more frequent potential transmission events that could result in subsequent infection of HCP and/or asymptomatic carriage and transmission to patients.^19^ In contrast with contact studies of other working adults, most HCP contacts are at work as opposed to outside of work, reinforcing the intensity of contact within the healthcare setting.^23^ Our study showed the relative stability of HCP contacts over time during the recovery from the COVID-19 pandemic, which has implications that it is difficult for HCP to reduce their risk of infection acquisition by contact number alone and rather must rely on other measures such as personal-protective equipment. The stability of contact also means that infection prevention or pandemic preparedness plans may be more generalizable across time or outbreak scenario.

Often during hospital outbreaks, staff on a given unit are the focus of interventions. These interventions depending on the pathogen may miss important HCP whose work spans multiple floors and could be potential targets for interventions such as transport workers or physical therapists. Additionally, it is notable that nearly 50% of HCP reported working despite the presence of symptoms and recent sick contact suggesting that they themselves may have been contagious with SARS-CoV-2 or other pathogens. The relatively greater frequency of contact with other HCP in the context of “presenteeism” supports policies encouraging or mandating vaccination of HCP against transmissible respiratory diseases and the universal use of respiratory personal protective equipment as source control during periods of transmission of respiratory pathogens.

Future work can incorporate more granular information such as patient-contact data to allow for social contact network analysis and or automated reporting such as sensors or badge recognition to obtain a more objective and complete analysis of HCP contacts. Additional work can investigate the role of HCP-to-HCP contact and further predict health care transmission of pathogens in transmission models. The importance of HCP-to-HCP contact should be considered in building design as well as improving ventilation of rooms where HCP congregate to reduce HAIs.

## Limitations

This study has several limitations. (1) This study is subject to selection bias as participation was voluntary. Particularly high-contact individuals would have less incentive to enroll or follow-up. (2) This study had a high loss to follow-up, likely reflecting the turnover in the hospital system. (3) Self-reported contact diaries are subject to recall bias and underreporting of the total number of contacts. However, participants did report that their overall contacts were mostly typical for their overall contact number, and the total number individual levels contacts matched 76% of the time with the summative contact numbers suggesting the total contacts was an adequate estimation. (4) Indirect contacts were lower than expected, perhaps indicating a recall bias. (5) There may be differential reporting by profession as certain jobs prioritize filling out forms or surveys accurately (such as social workers). (6) We lack the individual-level contact data for patients.

## Conclusions

Contact patters of HCP were relatively stable over time even during the recovery from COVID-19 suggesting less flexibility of HCP in limiting their number of contacts compared with other professions. HCP-to-HCP contact is a potential future area to explore for mitigating the transmission of healthcare-associated infections as HCP are 2.8 times more likely to have contact with other HCP than patients. Different HCP can be targeted in an individual outbreak depending on their job description and pathogen of interest, specifically HCP who span multiple units may be of particular interest to target given the quantity, intensity, and geographic diversity of their contacts.

## Supplementary Material

Supplemental_material

## Figures and Tables

**Table 1.** Participant Demographics: Participants demographics are described by participation area and completion of the contact diaries

Characteristic	Overall, N = 360^1^	Participation				Completed Intensive Diary
		Enrollment only, N = 168^1^	Longitudinal diaries, N = 95^1^	June 2022 diary, N = 35^1^	All activities, N = 62^1^	N = 88^1^
Age (years)	38 (30, 49)	37 (31, 48)	41 (30, 51)	33 (28, 44)	38 (31, 49)	36 (30, 50)
Gender						
Female	304 (84%)	141 (84%)	82 (86%)	27 (77%)	54 (87%)	75 (85%)
Male	55 (15%)	27 (16%)	12 (13%)	8 (23%)	8 (13%)	13 (15%)
Prefer not to answer	1 (0.3%)	0 (0%)	1 (1.1%)	0 (0%)	0 (0%)	0 (0%)
Race						
White or Caucasian	275 (76%)	121 (72%)	78 (82%)	30 (86%)	46 (74%)	70 (80%)
Asian	34 (9.4%)	17 (10%)	7 (7.4%)	3 (8.6%)	7 (11%)	10 (11%)
Black or African American	31 (8.6%)	20 (12%)	5 (5.3%)	1 (2.9%)	5 (8.1%)	4 (4.5%)
Other	20 (5.6%)	10 (6.0%)	5 (5.3%)	1 (2.9%)	4 (6.5%)	4 (4.5%)
Hispanic/Latino (Yes)	31 (8.6%)	17 (10%)	6 (6.3%)	3 (8.6%)	5 (8.1%)	8 (9.1%)
N household members						
1	69 (19%)	40 (24%)	14 (15%)	6 (17%)	9 (15%)	13 (15%)
2	115 (32%)	52 (31%)	29 (31%)	18 (51%)	16 (26%)	33 (38%)
3	61 (17%)	24 (14%)	20 (21%)	4 (11%)	13 (21%)	15 (17%)
4	69 (19%)	35 (21%)	16 (17%)	3 (8.6%)	15 (24%)	17 (19%)
5	30 (8.3%)	12 (7.1%)	10 (11%)	4 (11%)	4 (6.5%)	7 (8.0%)
6 or more	16 (4.4%)	5 (3.0%)	6 (6.3%)	0 (0%)	5 (8.1%)	3 (3.4%)
Job title						
Nurse	134 (37%)	70 (42%)	44 (46%)	7 (20%)	13 (21%)	19 (22%)
Doctor	69 (19%)	41 (24%)	15 (16%)	5 (14%)	8 (13%)	10 (11%)
Midlevel Provider	32 (8.9%)	15 (8.9%)	7 (7.4%)	3 (8.6%)	7 (11%)	9 (10%)
Other	27 (7.5%)	10 (6.0%)	10 (11%)	1 (2.9%)	6 (9.7%)	5 (5.7%)
Nursing Aid	21 (5.8%)	11 (6.5%)	6 (6.3%)	0 (0%)	4 (6.5%)	4 (4.5%)
Administrative staff	15 (4.2%)	5 (3.0%)	2 (2.1%)	6 (17%)	2 (3.2%)	8 (9.1%)
Diagnostic Imaging Technologist	13 (3.6%)	3 (1.8%)	3 (3.2%)	2 (5.7%)	5 (8.1%)	7 (8.0%)
Laboratory	11 (3.1%)	1 (0.6%)	0 (0%)	3 (8.6%)	7 (11%)	10 (11%)
Rehabilitation/transport	11 (3.1%)	2 (1.2%)	4 (4.2%)	2 (5.7%)	3 (4.8%)	4 (4.5%)
Pharmacist	7 (1.9%)	2 (1.2%)	0 (0%)	3 (8.6%)	2 (3.2%)	5 (5.7%)
Social Worker	7 (1.9%)	2 (1.2%)	2 (2.1%)	1 (2.9%)	2 (3.2%)	3 (3.4%)
Dietician	6 (1.7%)	1 (0.6%)	0 (0%)	2 (5.7%)	3 (4.8%)	4 (4.5%)
Respiratory Therapist	5 (1.4%)	3 (1.8%)	2 (2.1%)	0 (0%)	0 (0%)	0 (0%)
Researcher	2 (0.6%)	2 (1.2%)	0 (0%)	0 (0%)	0 (0%)	0 (0%)
Department						
Primary Care/Internal Medicine/Pediatrics	152 (42%)	71 (42%)	42 (44%)	11 (31%)	28 (45%)	34 (39%)
Emergency Medicine	55 (15%)	39 (23%)	8 (8.4%)	3 (8.6%)	5 (8.1%)	7 (8.0%)
Other	89 (25%)	38 (23%)	29 (31%)	11 (31%)	11 (18%)	21 (24%)
Surgery	31 (8.6%)	12 (7.1%)	12 (13%)	3 (8.6%)	4 (6.5%)	7 (8.0%)
Laboratory	17 (4.7%)	6 (3.6%)	1 (1.1%)	4 (11%)	6 (9.7%)	10 (11%)
Rehab/transport	9 (2.5%)	1 (0.6%)	2 (2.1%)	1 (2.9%)	5 (8.1%)	4 (4.5%)
Radiology	7 (1.9%)	1 (0.6%)	1 (1.1%)	2 (5.7%)	3 (4.8%)	5 (5.7%)
Years working as HCP						
10 years or less	182 (51%)	91 (54%)	41 (43%)	22 (63%)	28 (45%)	45 (51%)
11 to 20 years	92 (26%)	42 (25%)	25 (26%)	8 (23%)	17 (27%)	22 (25%)
20 years or more	86 (24%)	35 (21%)	29 (31%)	5 (14%)	17 (27%)	21 (24%)
Average number of hours worked per week						
0-20 hours per week	13 (3.6%)	4 (2.4%)	8 (8.5%)	0 (0%)	1 (1.6%)	0 (0%)
21-40 hours per week	292 (82%)	135 (81%)	68 (72%)	33 (94%)	56 (90%)	81 (92%)
41-60 hours per week	46 (13%)	23 (14%)	16 (17%)	2 (5.7%)	5 (8.1%)	7 (8.0%)
60 hours per week or more	7 (2.0%)	5 (3.0%)	2 (2.1%)	0 (0%)	0 (0%)	0 (0%)
History of COVID-19 at time of enrollment						
No	264 (74%)	118 (72%)	77 (81%)	23 (66%)	46 (74%)	63 (72%)
Yes, and it was confirmed by a diagnostic test	82 (23%)	41 (25%)	13 (14%)	12 (34%)	16 (26%)	25 (28%)
Yes, but it was not confirmed by a diagnostic test	11 (3.1%)	6 (3.6%)	5 (5.3%)	0 (0%)	0 (0%)	0 (0%)
Vaccinated for COVID-19 at the time of enrolment (Yes)	287 (81%)	121 (74%)	71 (75%)	34 (97%)	61 (98%)	86 (98%)

1 Median (IQR); n (%).

**Table 2.** Total number of contacts and contacts with patients and HCP by job January 2021–June 2022: Total number of contacts by job was tested via negative binomial regression. Ordinal variables for average direct contacts with patients, average time spent with patients, and average direct contacts with healthcare personnel (HCP) were tested with a cumulative linked mixed model. The reference group was the job with the greater percentage of response in the lowest category for that contact type

Jobs	Total N contacts		Average direct contacts with patients		Average time spent with patients		Average direct contacts with HCP	
	exp (Beta)	95% CI^1^	log (OR)^1^	95% CI^1^	log (OR)^1^	95% CI^1^	log (OR)^1^	95% CI^1^
Administrative staff	1.2	0.64, 2.24	0.96	−1.5, 3.4	−19	−430, 392	−0.5	−2.1, 1.1
Diagnostic Imaging Technologist	2.22	1.20, 4.08	5.8	4.3, 7.3	7.4	4.1, 11	0.12	−1.5, 1.7
Dietician	1.82	0.86, 3.82	5.9	3.7, 8.1	5.6	2.1, 9.1	−1.2	−3.3, 0.86
Doctor	1.7	1.03, 2.80	6.1	5.2, 7.0	5.3	2.4, 8.1	0.02	−0.95, 0.98
Laboratory	2.03	1.11, 3.71	—	—	—	—	1.2	−0.45, 2.8
Midlevel Provider	2.06	1.21, 3.54	4.4	4.4, 4.4	5.6	2.7, 8.6	0.24	−0.94, 1.4
Nurse	1.95	1.22, 3.11	3.8	3.1, 4.5	5.8	3.0, 8.6	—	—
Nursing Aid	—	—	5.7	4.1, 7.2	6.3	3.1, 9.5	−0.59	−2.1, 0.89
Other	1.69	0.94, 3.04	4	2.4, 5.6	3.3	0.19, 6.4	−0.02	−1.5, 1.4
Pharmacist	1.45	0.67, 3.12	4.1	1.4, 6.7	3.3	−0.30, 7.0	1.2	−0.92, 3.2
Rehabilitation/transport	3.07	1.60, 5.91	6.8	5.0, 8.6	9.7	6.0, 13	0.61	−1.1, 2.4
Respiratory Therapist	2.07	0.78, 5.51	7.4	4.3, 11	4.3	−0.22, 8.9	−0.05	−3.1, 3.0
Social Worker	1.2	0.60, 2.43	5.5	3.4, 7.6	6.2	2.8, 9.6	0.03	−1.8, 1.9
Time spline	1.27	0.99, 1.64	0.84	0.83, 0.85	−0.61	−1.6, 0.37	0.06	−0.81, 0.93

1 OR = odds ratio, CI = confidence interval.
