# Supplementary material for: Social mixing patterns of United States healthcare personnel at a quaternary health center: a prospective observational study
Source: Infect Control Hosp Epidemiol. Author manuscript; Available in PMC 2025 Mar 7. (PMC11883655; doi:10.1017/ice.2024.234)
Supplement: Supplemental_material [file NIHMS2056795-supplement-Supplemental_material.docx]

Table of Contents

[Supplementary Table 1: Additional definitions and study details methods 2](#_Toc174539813)

[Supplementary Table 2: Enrolment survey questions 3](#_Toc174539814)

[Supplementary Table 3: Variables from the monthly longitudinal contact diary 9](#_Toc174539815)

[Supplemental Table 4: Variables from Intensive 2-day contact diary 15](#_Toc174539816)

[Supplemental Table 3: STROBE Statement—Checklist of items that should be included in reports of cohort studies 22](#_Toc174539817)

[Supplemental Table 4: Demographics cont. 24](#_Toc174539818)

[Supplemental Table 5: Health care personnel contacts over time 26](#_Toc174539819)

[Supplemental Table 6: Detailed contact characteristics from longitudinal contact diaries (January 2021-May 2022) and intensive 2-day contact diary, June 2022 32](#_Toc174539820)

[Supplemental Table 7: Detailed contact characteristics from intensive 2-day contact diary, June 2022 39](#_Toc174539821)

[Supplemental Figure 1: Geographic of primary hospital lay out 41](#_Toc174539822)

[Supplemental Figure 2: Age-contact matrices by locations and other individuals 42](#_Toc174539823)

[Supplemental Figure 3: Age-contact matrices by contact relationship (patient vs HCP) and contact type (direct proximity, non-physical, physical contact) 43](#_Toc174539824)

[Supplemental Figure 4: Detailed vs. summative reported contacts in June 2022 intensive contact diary 44](#_Toc174539825)

Supplementary Table 1: Additional definitions and study details methods

***Primary hospital:*** The primary hospital of enrollment was a quaternary hospital with 1,541 beds with over 100,000 emergency room visits per year that is split across 2 campuses (campus 1 and campus 2). Campus 1 had 4 medical towers, a laboratory building, emergency department (ED) and several adjacent clinics. Campus 2 was several blocks away and serves as a general hospital. Clinics or other hospitals beyond these two footprints were characterized as offsite facilities.

***Additional survey methods:*** An optional paper-contact diary was offered to participants that they could submit to study staff who would manually enter this information into Qualtrics. Initially the monthly contact diary collected individual-level contact information but due to poor response rates, the study paused June 2021 to July 2021 to update and simplify the monthly contact diaries to summative contacts.

***Definitions:***

**Large group** **gatherings:** where participants had contact with others but could not identify everyone individually during the reported day

# Supplementary Table 2: Enrolment survey questions

| **Variable name** | **Variable Description** | **Type** | **Levels/Notes** |
| --- | --- | --- | --- |
| Q8 | Please indicate below the unit(s) in which you work. Please select all that apply. | Nominal | - Unit 1 (unit names coded in manuscript) - Unit 2 - Unit 3 - Unit 4 (ICU) - Unit 5 - Other (Free Text) - Emergency Department |
| Q10 | Please enter your date of birth (mm/dd/yyyy) below. | Date |  |
| Q11 | Gender | Nominal | - Female - Male - Non-binary - Prefer not to answer |
| Q12 | What is the highest level of education you have completed? | Ordinal | - Less than high school degree - High school graduate (or GED equivalent) - Some college but no degree - Associate degree in college - Bachelor’s degree in college - Master’s degree - Doctoral Degree - Technical License |
| Q13 | How many years of education have you completed since high school graduation? | Ordinal | - Less than 5 years - 5-10 years - 11-15 years - 16-20 years - More than 20 years |
| Q14 | Country of birth | Nominal | List of countries |
| Q23 | What state do you live in | Nominal | List of all 50 states |
| Q15 | Race | Nominal | - American Indian/Alaska Native - Asian - Black or African American - Native Hawaiian or other Pacific Islander - White - Other - Prefer not to say |
| Q16 | Hispanic, Latinx or Spanish in origin | Nominal | - Yes   - Mexican, Mexican American, Chicano   - Puerto Rican   - Cuban   - Another Hispanic, Latinx or Spanish origin   - Prefer not to say - No |
| Q17 | Marital status | Nominal | - Single - Married - Widowed - Divorced - Separated - Cohabitating with partner |
| Q18 | Who do you live with? Please select all that apply | Nominal | - Cohabitating - Roommates - I live alone - Parents - Other, please specify: - Children - Siblings |
| Q19 | How many people are currently living or staying at your home, including yourself? | Ordinal | - 1 - 2 - 3 - 4 - 5 - 6 or more |
| Q20 | How many of these individuals are 18 years old or older, including yourself? | Ordinal | - 1 - 2 - 3 - 4 - 5 - 6 or more |
| Q24 | What is your job category | Nominal | - Administrative staff - Attending physician (please specify specialty) - Diagnostic imaging technologist - Emergency medical technician - Midlevel Provider (APRN/PA) - Nurse (RN/BSN/MSN) - Nursing Aid (PCT/CNA/LPN etc.) - Patient attendant/sitter - Pharmacist - Phlebotomist - Physical Therapist/Occupational Therapist - Resident Physician - Postgraduate medical Trainee - Researcher - Respiratory Therapist - Social Worker - Support staff (e.g., dietary aides, environmental services, transport etc.) - Unit Secretary - Other, please specify - Student, please specify |
| Job_sum | Summarized from Q24 above | Nominal | Participant’s job was summarized as within 12 categories: Administrative staff, Dietician, Doctor, Diagnostic imaging technologist, Laboratory, Midlevel provider, Nursing aid, Other, Pharmacist, Rehabilitation/transport, social worker |
| Q25 | In which of the following departments do you work? Please select all that apply. | Nominal | - Anesthesiology - Cardiovascular medicine - Digestive Diseases - Endocrine and metabolism - Emergency Medicine - General Medicine - Geriatrics - Hematology - Infectious Diseases - Medical Oncology - Nephrology - Palliative Care - Pubic Health - Pulmonary, Critical Care, and Sleep Medicine - Primary Care (OB/Gyn, Family Medicine, Paediatrics) - Rheumatology, Allergy and Immunology - Radiology - Surgery - Department of Internal Medicine (Dept Level Staff) - Internal Medicine Hospital Residents - Other, please specify |
| Q26 | How many years of experience do you have as a healthcare worker? | Ordinal | - Less than 5 years - 6-10 years - 11-15 years - 16-20 years - More than 20 years |
| Q27 | What is your annual household income before taxes | Ordinal | - Less than $20,000 - $20,000 to $34,999 - 35,000 to $49,999 - 50,000 to $74,999 - 75,000 to $99,999 - More than 100,000 |
| Q21 | What best describes the house you currently live in? | Nominal | - Detached house - Townhouse - Condominium - Other, please specify - Apartment |
| Q22 | How many units are in your condominium/apartment? | Nominal | - < 50 units - 50-99 units - 100-199 units - 200 units |
| Q28 | On average, how many hours do you work at the hospital per week? | Ordinal | - 0-20 hours per week - 21-40 hours per week - 40-60 hours per week - More than 60 hours per week |
| Q29 | What chronic medical conditions have you been diagnosed with? Please select all that apply. | Nominal | - Asthma or lung disease - Cancer - Congenital disease - COPD - Diabetes Mellitus - Genetic disease - Heart Disease - Hematologic diseases - Hypertension - Kidney disease - Liver disease - Neurological diseases - Stroke - Other, please specify - Pregnant - None |
| Q37 | Have you ever consumed any of the following | Nominal | - Alcohol - Tobacco - Recreational drugs |
| Q32 | Have you ever gotten the yearly influenza vaccine? | Ordinal | - Yes, every year - Yes, but not every year - No, never |
| Q33 | When was your last flu shot? | Ordinal | - August 2017-March 2018 - August 2018-March 2019 - August 2019-March 2020 - August 2020-March 2021 - After August 2021 |
| Q34 | Have you had COVID-19 | Ordinal | - Yes, and it was confirmed by a diagnostic test - Yes, but it was not confirmed by a diagnostic test   No, never |
| Q35 | Have you been vaccinated against COVID-19? | Binary | - Yes - No |
| Q36 | Have you ever participated in telecommuting/teleworking? Telecommute/telework is defined as work from a location outside of your designated place of work. It may consist of working from home, from a public space outside of your workplace, etc. | Binary | - Yes   No |
| Q37 | How often did you telecommute/telework before the COVID-19 pandemic? | Ordinal | - Every day - Multiple times a week - Once a week - Multiple times a month - Once a month - Less than once a month - Never |
| Q38 | Where do you primarily work from when you telecommute/telework? | Nominal | - Home - Coffee shop - Other, please specify - Co-working space - Restaurant |
| Q40 | What form of transit do you use to get to this telecommute/telework location? Please select all that apply. | Nominal | - None - Walking or biking - Driving yourself - Carpool - Rideshare - Public transport - Other please specify |

# Supplementary Table 3: Variables from the monthly longitudinal contact diary

| **Variable name** | **Variable Description** | **Type** | **Levels/Notes** |
| --- | --- | --- | --- |
| Q2 | Please enter the unit number you worked in for the date you are reporting contacts for: | Nominal | - Free text |
| Q2_6_TEXT | Please  enter the unit number you worked in for the date you are reporting contacts for: Other | Nominal | - Entered as free text |
| sub_floor | Created from Q2, Q2_6_TEXT, 1_Q25 to 1_Q27 | Nominal | Individuals entered the unit where they worked and this was categorized as sub-floor, floor, building, hospital and an overall location of inpatient or outpatient. If participants worked across multiple buildings in the hospital, their building was denoted as the overall hospital. If participants worked across multiple buildings beyond the hospital footprint, these were classified as “multi” location. If participants worked both as an inpatient and outpatient, they were classified as inpatient. |
| building | Created from Q2 and Q2_6_TEXT, 1_Q25 to 1_Q27 | Nominal | Building number reported by HCP, see note for sub_floor |
| building_sum | Created from Q2 and Q2_6_TEXT, 1_Q25 to 1_Q27 | Nominal | Building summary number reported by HCP   - Hospital 1 - Hospital 2   Off site |
| in_out | Created from Q2 and Q2_6_TEXT, 1_Q25 to 1_Q27 | Nominal | Inpatient vs outpatient summary number reported by HCP   - Inpatient - Outpatient |
| Q4 | Please enter the date you are reporting contacts for: (mm/dd/yyyy) | Date | N/A |
| Q5 | What day of the week are you reporting contacts for? | Nominal | Monday to Sunday |
| Q6 | In the past one month, have you experienced any of the following symptoms? (Select all that apply.) - Selected Choice | Nominal | - Objective fever (>100.4 F) - Subjective fever - Cough (new onset or worsening of cough) - Dyspnea/Shortness of Breath - Runny nose - Sore throat - Unusually hoarse voice - Persistent chest pain - Chest discomfort - Chills - Sweating - Malaise - Fatigue - Muscle aches/pain - Loss of appetite - Nausea - Vomiting - Diarrhea - Abdominal pain - Headache - Dizziness - Loss of taste - Loss of smell - Sneezing - Skin rashes - None of these - Other (please describe) |
| Q6_24_TEXT | In the past one month, have you experienced any of the following symptoms? – Other (please describe). | Nominal | - Entered as free text |
| Q7 | On average, how long was the duration of the most recent episode of illness? | Ordinal | - Few hours - 1 day - 2 to 7 days - 1 to 2 weeks - More than 2 weeks   None of these |
| Q8 | Did you show up for work at any work location while having this episode of illness? | Binary | - Yes - No |
| Q9 | In the past one month, did any member of your household also experience any of these symptoms? | Nominal | - Yes - No - Not sure - Does not apply to me |
| Q10 | In the past 1 month, how many shifts have you worked on a COVID-19 unit? | Ordinal | - None - 1-5 shifts - 6-10 shifts - 11-15 shifts - 16-20 shifts - >20 shift |
| Q11 | In room with confirmed COVID-19 patient during a Code Blue | Nominal | - Yes - No - I don’t know |
| Q12 | How many times were you in the room with a confirmed COVID-19 patient during a code blue (or the equivalent of a code blue in the Emergency Department)? | Ordinal | - 0 - 1-5 - 6-10 - 11-15 - 16-20 - >20 |
| Q13 | Did you participate in chest compressions during a Code Blue with a confirmed COVID-19 patient | Binary | - Yes - No |
| Q14 | Did you participate in medication administration during a Code Blue with a confirmed COVID-19 patient | Binary | - Yes - No |
| Q15 | Did you participate in auscultation during a code blue with a confirmed COVID-19 patient? | Binary | - Yes - No |
| Q16 | In the past 1 month, were you within 6 feet of a confirmed COVID-19 patient during an intubation procedure? | Nominal | - Yes - No - I don’t know |
| Q17 | How many times were you within 6 feet of a confirmed COVID-19 patient during an intubation process procedure? | Ordinal | - 1-5 - 6-10 - 11-15 - 16-20 - >20 |
| Q18 | If you were within 6 feet of a confirmed COVID-19 patient during an intubation procedure, which of the following did you wear? (Select all that apply) | Nominal | - N95 - Surgical face mask - PAPR - Other (P100) |
| Q19 | Approximately, how many patients were you in direct contact (within 6 feet) with on this day? | Ordinal | - Initially <5, 5-9, 10-19, 20-29, 30-39, 40-49, > 50 patients - compressed to <5, 5-9, 10-19, ≥ 20 patients in analysis |
| Q20 | On average, how many minutes did you spend in direct contact with each patient (within 6 feet) on this day? | Ordinal | - 5, 5-10, 11-20, >20 minutes |
| Q21 | On average, with how many patients did you have indirect contact (same room, but not closer than 6 feet) on this day? | Ordinal | - <5, 6-10, 11-15, 16-20, 21-25, 26-30, >30 patients - compressed to <5, 6-10, 11-15, 16-20, >20 in analysis |
| Q22 | On average, with how many healthcare co-workers (HCP) did you have direct contact with (within 6 feet for at least two minutes, no glass or wall partition separating you) on this day? | Ordinal | - <5, 6-10, 11-15,16-20, 21-25, 26-30, > 30 co-workers; compressed to <5, 6-10, 11-15,16-20, >20 in analysis |
| Q23_1 | On a scale from 1-5, how would you rate this week's contacts compared to a typical week? 1 being not at all typical, 5 being extremely typical. | Ordinal | - Scale 1 to 5 |
| Q30_1 | Please enter how many different physical locations in terms of hospital, building, floor, unit you worked in on this day.  For example, if you worked in floor 1 and 2 of building "A", you would enter 2. Or if you worked in the Delivery Unit and NICU in the same building, you would enter 2. | Discrete | - Entered as integer |
| 1_Q25 | 1 - Please name the hospital in which you worked on the day you are reporting for. If you worked at more than one, please only choose one. | Nominal | - This question was repeated as many times as listed in Q30_1 - Free text |
| 1_Q26 | 1 - Please enter the name of the building you worked in at this hospital. | Nominal | - This question was repeated as many times as listed in Q30_1 - Free text |
| 1_Q27 | 1 - Please enter the name of the floor you worked in this building. | Nominal | - This question was repeated as many times as listed in Q30_1 - Free text |
| 1_Q28 | Please enter the unit number you worked in for the date you are reporting contacts for: - 1 - Other, please specify: - Text | Nominal | - This question was repeated as many times as listed in Q30_1 - Free text |
| Q29 | 1 - Approximately, how many hours did you work in this unit today? | Discrete | - This question was repeated as many times as listed in Q30_1 - Entered as integer |
| Q30 | Were you in any large group gathering (e.g. airport, bus stations, train stations, meetings, grocery store, hospitals, public performances, movie theaters, etc.) where you had contact with others, but could not identify everyone individually?     As a reminder, contact can be defined as:   Physical contact: directly touching someone (skin-to-skin contact), or the clothes they are wearing intentionally or unintentionally (for example, a handshake, fist bump, elbow bump, foot bump, hug, kiss, etc.)  Non-physical contact: a two-way conversation with three or more words exchanged in the physical presence (within 2 m or approximately 6 ft of each other) but with no physical contact. Direct proximity: being within 6 feet of your contact for 20 seconds or more with neither conversation nor physical contact. | Binary | - Yes - No |
| Q31 | How many people did you come into contact with (as defined in the previous question) on the day you are evaluating for? Please exclude people you could not identify in large groups. | Discrete | - Entered as integer |

# Supplemental Table 4: Variables from Intensive 2-day contact diary

Q6 to Q18 were the same as Supplemental Table 1, with the exception that the reported time period was 14 days as opposed to 1 month. Q21 to Q28 were repeated twice, once per each day reported.

| **Variable** | **Variable Description** | **Type** | **Levels/Notes** |
| --- | --- | --- | --- |
| Q2_1 | Enter a date: - Day 1 | Date | mm/dd/yyyy |
| Q2_2 | Enter a date: - Day 2 | Date | mm/dd/yyyy |
| Q21/Q62 | Approximately, how many patients were you in direct contact (within 6 feet) with on day 1 (Q21)/ day 2 (Q62)? | Ordinal | - Initially <5, 5-9, 10-19, 20-29, 30-39, 40-49, > 50 patients - compressed to <5, 5-9, 10-19, ≥ 20 patients in analysis |
| Q22/Q63 | On average, how many minutes did you spend in direct contact with each patient (within 6 feet) on day 1 (Q22)/ day 2 (Q63)? | Ordinal | - 5 minutes, - 5-10 minutes, - 11-20 minutes, - >20 minutes |
| Q23/Q64 | On average, with how many patients did you have indirect contact (same room, but not closer than 6 feet) on day 1 (Q23)/ day 2 (Q64)? | Ordinal | - <5, 6-10, 11-15, 16-20, 21-25, 26-30, >30 patients - compressed to <5, 6-10, 11-15, 16-20, >20 in analysis |
| Q24/Q65 | On average, with how many healthcare co-workers (HCP) did you have direct contact with (within 6 feet for at least two minutes, no glass or wall partition separating you) on day 1 (Q24)/ day 2 (Q 64)? | Ordinal | - <5, 6-10, 11-15,16-20, 21-25, 26-30, > 30 co-workers - compressed to <5, 6-10, 11-15,16-20, >20 in analysis |
| Q25/Q66 | On a scale from 1-5, how would you rate this week's contacts compared to a typical week? 1 being not at all typical, 5 being extremely typical. Day 1 Q25/ day 2 Q66) | Ordinal | - Scale 1 to 5 |
| Q26/Q67 | Please enter the number of different physical locations you worked on day 1 (Q26)/Day 2 (Q67).  Physical locations include all of the hospitals, buildings, floors, units that you worked in. For example, if you worked in floor 1 and 2 of building "A", you would enter 2. Or if you worked in the Delivery Unit and NICU in the same building, you would enter 2. | Discrete | - Entered as integer |
| Q27/Q68 | Please enter the number of hospitals you worked in on day 1(Q27)/day 2 (Q68) | Discrete | - Entered as integer |
| Q28/Q69 | What is the name of the hospital which you worked the most hours on day 1 (Q28)/ day 2 (Q69)? | Nominal | - Entered as free text |
| Q19/Q60 | Were you in any large group gathering (e.g. airport, bus stations, train stations, meetings, grocery store, hospitals, public performances, movie theaters, hospital cafeteria, etc.) where you had contact with others, but could not identify everyone individually on day 1 (Q19)/ day 2 (Q60)?     As a reminder, contact can be defined as:   Physical contact: directly touching someone (skin-to-skin contact), or the clothes they are wearing intentionally or unintentionally (for example, a handshake, fist bump, elbow bump, foot bump, hug, kiss, etc.)  Non-physical contact: a two-way conversation with three or more words exchanged in the physical presence (within 2 m or approximately 6 ft of each other) but with no physical contact. Direct proximity: being within 6 feet of your contact for 20 seconds or more with neither conversation nor physical contact. | Binary | - Yes - No |
| Q20/Q61 | How many people including patients, coworkers, family, etc. did you come into contact with (as defined in the previous question) on day 1 (Q20)/ day 2 (Q61)? Please exclude people you could not identify in large groups (e.g. YNHH emergency room). | Nominal | - Entered as integer |
| 1_Q31/1_Q72 | Please name the hospital in which you worked on the day you are reporting for. If you worked at more than one, please only choose one. Day 1(1_Q31)/ Day 2 1_Q72 | Nominal | This question was repeated as many times as listed in Q26/Q67   - Free text - Used to created variables sub_floor, building, building_sum and in_out (see supplemental table 1) |
| 1_Q32/1_Q73 | 1 - Please enter the name of the building you worked in at this hospital. Day 1 (1_Q32) Day 2 (1_Q73) | Nominal | This question was repeated as many times as listed in Q26/Q67   - Free text - Used to created variables sub_floor, building, building_sum and in_out (see supplemental table 1) |
| 1_Q33/1_Q74 | Please enter the name of the floor you worked in this building. Day 1 (1_Q33) / Day 2 (1_Q74) | Nominal | This question was repeated as many times as listed in Q26/Q67   - Free text - Used to created variables sub_floor, building, building_sum and in_out (see supplemental table 1) |
| 1_Q33_1/  1_Q74_1 | Please enter the name of the floor you worked in this building. Day 1 (1_Q33_1)/ Day 2 (1_Q74_1) | Nominal | This question was repeated as many times as listed in Q26/Q67   - Free text - Used to created variables sub_floor, building, building_sum and in_out (see supplemental table 1) |
| 1_Q33_0/  1_Q74_1 | Please enter the name of the sub-floor you worked in this building. Day 1 (1_Q33_0)/ Day 2 (1_Q74_0) | Nominal | This question was repeated as many times as listed in Q26/Q67   - Free text - Used to created variables sub_floor, building, building_sum and in_out (see supplemental table 1) |
| 1_Q34/1_Q75 | Approximately, how many hours did you work in this floor on day 1 (1_Q34) Day 2 (1_Q75)? | Discrete | This question was repeated per number of contacts reported   - Entered as integer |
| 1_Q37/1_Q78 | Please enter a unique identifier for the person you are currently reporting on.  This can be anything that would help you remember this person tomorrow, such as nicknames (i.e., lady in red hat, Nurse 1, first name, etc.). Day 1 (1_Q37) Day 2 (1_Q78) | Nominal | This question was repeated per number of contacts reported   - Free text |
| 1_Q38/  1_Q81 | What is your relationship with this person?  Day 1 (1_Q38) / Day 2  (1_Q81) | Nominal | This question was repeated per number of contacts reported   - household member - relative - friend/acquaintance - colleague, patient - do not know this person personally and not a patient |
| 1_Q39/  1_Q82 | What is this person's gender? Day 1 (1_Q39) / Day 2(1_Q82) | Nominal | This question was repeated per number of contacts reported   - female - male - non-binary - unknown |
| 1_Q40/  1_Q83 | Please choose the estimated age range of this person. Day 1 (1_Q40) / Day 2 (1_Q83) | Ordinal | This question was repeated per number of contacts reported   - 0-1, - 1–9, - 10–19, - 20–29, - 30–39, - 40–49, - 50-59, - 60-69, - 70-79, - 80+ |
| 1_Q41/  1_Q84 | Did you make physical contact and/or speak with this person? Please select all that apply. Day 1 (1_Q41) / Day 2 (1_Q84) | Nominal | This question was repeated per number of contacts reported   - Non-physical contact: a two-way conversation with three or more words exchanged in the physical presence (within 2 m or approximately 6 ft of each other) but with no physical contact - Non-physical contact: a two-way conversation with three or more words exchanged in the physical presence (within 2 m or approximately 6 ft of each other) but with no physical contact - Direct proximity: being within 6 feet of your contact for 20 seconds or more with neither conversation nor physical contact |
| 1_Q42/  1_Q85 | Where did you come into contact with this person? Please select all that apply. - 1 - Where did you come into contact with this person? Please select all that apply. - Selected Choice Day 1(1_Q42)/ Day 2 (1_Q85) | Nominal | This question was repeated per number of contacts reported   - hospital - emergency room - ambulatory setting - home - other work associated - other non-work associated |
| 1_Q43/  1_Q86 | What was the total time you spent with this person during the entire day (including overnight)? Day 1 (1_Q43) Day 2 (1_Q86) | Ordinal | This question was repeated per number of contacts reported   - less than 5 minutes - 5-15 minutes - 5 minutes-1hour - 1-4 hours - more than 4 hours |
| 1_Q44/  1_Q87 | How often do you have contact with this person, in general?  Day 1 (1_Q44) / Day 2 (  1_Q87) | Ordinal | This question was repeated per number of contacts reported   - daily or almost daily - 1-2 times per week - 1-2 times per month - less than once per month - no contact in general |
| 1_Q45/  1_Q88 | 1 - Was this contact wearing one of our study proximity sensors? Day 1 (1_Q45) / Day 2  (1_Q88) | Nominal | This question was repeated per number of contacts reported   - Yes - No - Unsure |
| 1_Q79 | Did you have contact with and record this person on your first day? | Binary | This question was repeated per number of contacts reported   - Yes - No |
| 1_Q80 | What is the identifier you used for this person on Day 1? | Nominal | This question was repeated per number of contacts reported  Free text |

# Supplemental Table 5: STROBE Statement—Checklist of items that should be included in reports of *cohort studies*

|  | Item No | Recommendation | Page No |
| --- | --- | --- | --- |
| **Title and abstract** | 1 | (*a*) Indicate the study’s design with a commonly used term in the title or the abstract | 1-2 |
|  |  | (*b*) Provide in the abstract an informative and balanced summary of what was done and what was found | 2 |
| Introduction | | | |
| Background/rationale | 2 | Explain the scientific background and rationale for the investigation being reported | 3 |
| Objectives | 3 | State specific objectives, including any prespecified hypotheses | 3 |
| Methods | | | |
| Study design | 4 | Present key elements of study design early in the paper | 4-5 |
| Setting | 5 | Describe the setting, locations, and relevant dates, including periods of recruitment, exposure, follow-up, and data collection | 4, ST1 |
| Participants | 6 | (*a*) Give the eligibility criteria, and the sources and methods of selection of participants. Describe methods of follow-up | 4 |
|  |  | (*b*) For matched studies, give matching criteria and number of exposed and unexposed | NA |
| Variables | 7 | Clearly define all outcomes, exposures, predictors, potential confounders, and effect modifiers. Give diagnostic criteria, if applicable | 5 |
| Data sources/ measurement | 8* | For each variable of interest, give sources of data and details of methods of assessment (measurement). Describe comparability of assessment methods if there is more than one group | ST2-4 |
| Bias | 9 | Describe any efforts to address potential sources of bias | 5 |
| Study size | 10 | Explain how the study size was arrived at | 5 |
| Quantitative variables | 11 | Explain how quantitative variables were handled in the analyses. If applicable, describe which groupings were chosen and why | 5 |
| Statistical methods | 12 | (*a*) Describe all statistical methods, including those used to control for confounding | 6 |
|  |  | (*b*) Describe any methods used to examine subgroups and interactions | 6 |
|  |  | (*c*) Explain how missing data were addressed | 6 |
|  |  | (*d*) If applicable, explain how loss to follow-up was addressed | 6 |
|  |  | (*e*) Describe any sensitivity analyses |  |
| Results | | |  |
| Participants | 13* | (a) Report numbers of individuals at each stage of study—e.g. numbers potentially eligible, examined for eligibility, confirmed eligible, included in the study, completing follow-up, and analyzed | 7 |
|  |  | (b) Give reasons for non-participation at each stage | 7 |
|  |  | (c) Consider use of a flow diagram | - |
| Descriptive data | 14* | (a) Give characteristics of study participants (e.g. demographic, clinical, social) and information on exposures and potential confounders | 7, T1, ST6 |
|  |  | (b) Indicate number of participants with missing data for each variable of interest | T1, ST5 |
|  |  | (c) Summarise follow-up time (eg, average and total amount) | 7 |
| Outcome data | 15* | Report numbers of outcome events or summary measures over time | - |

| Main results | 16 | (*a*) Give unadjusted estimates and, if applicable, confounder-adjusted estimates and their precision (e.g., 95% confidence interval). Make clear which confounders were adjusted for and why they were included | 8 |
| --- | --- | --- | --- |
|  |  | (*b*) Report category boundaries when continuous variables were categorized |  |
|  |  | (*c*) If relevant, consider translating estimates of relative risk into absolute risk for a meaningful time period |  |
| Other analyses | 17 | Report other analyses done—e.g., analyses of subgroups and interactions, and sensitivity analyses | 9 |
| Discussion | | | |
| Key results | 18 | Summarise key results with reference to study objectives | 9-10 |
| Limitations | 19 | Discuss limitations of the study, taking into account sources of potential bias or imprecision. Discuss both direction and magnitude of any potential bias | 11 |
| Interpretation | 20 | Give a cautious overall interpretation of results considering objectives, limitations, multiplicity of analyses, results from similar studies, and other relevant evidence | 10-12 |
| Generalisability | 21 | Discuss the generalisability (external validity) of the study results | 11 |
| Other information | | | |
| Funding | 22 | Give the source of funding and the role of the funders for the present study and, if applicable, for the original study on which the present article is based | 12 |

*Give information separately for exposed and unexposed groups.

**Note:** An Explanation and Elaboration article discusses each checklist item and gives methodological background and published examples of transparent reporting. The STROBE checklist is best used in conjunction with this article (freely available on the Web sites of PLoS Medicine at http://www.plosmedicine.org/, Annals of Internal Medicine at http://www.annals.org/, and Epidemiology at http://www.epidem.com/). Information on the STROBE Initiative is available at http://www.strobe-statement.org.

# Supplemental Table 6: Demographics continued

|  | **Participation** | | | | | **Completed Intensive Diary** |
| --- | --- | --- | --- | --- | --- | --- |
| **Characteristic** | **Overall**, N = 360^1^ | **Enrollment only**, N = 168^1^ | **Longitudinal diaries**, N = 95^1^ | **Sensor week diary**, N = 35^1^ | **All activities**, N = 62^1^ | **N = 88**^1^ |
| Education |  |  |  |  |  |  |
| High school graduate or GED | 9 (2.5%) | 5 (3.0%) | 1 (1.1%) | 1 (2.9%) | 2 (3.2%) | 3 (3.4%) |
| Technical license | 6 (1.7%) | 3 (1.8%) | 2 (2.1%) | 0 (0%) | 1 (1.6%) | 1 (1.1%) |
| Some college but no degree | 18 (5.0%) | 7 (4.2%) | 4 (4.2%) | 1 (2.9%) | 6 (9.7%) | 6 (6.8%) |
| Associate's degree | 26 (7.2%) | 15 (8.9%) | 7 (7.4%) | 0 (0%) | 4 (6.5%) | 4 (4.5%) |
| Bachelor's degree | 115 (32%) | 52 (31%) | 34 (36%) | 11 (31%) | 18 (29%) | 28 (32%) |
| Master's degree | 96 (27%) | 40 (24%) | 24 (25%) | 13 (37%) | 19 (31%) | 29 (33%) |
| Doctoral degree | 90 (25%) | 46 (27%) | 23 (24%) | 9 (26%) | 12 (19%) | 17 (19%) |
| Country of birth |  |  |  |  |  |  |
| United States of America | 307 (85%) | 141 (84%) | 82 (86%) | 32 (91%) | 52 (84%) | 76 (86%) |
| Outside of United States | 53 (15%) | 27 (16%) | 13 (14%) | 3 (8.6%) | 10 (16%) | 12 (14%) |
| Marital status |  |  |  |  |  |  |
| Married or domestic partnership | 207 (58%) | 86 (51%) | 59 (62%) | 24 (69%) | 38 (61%) | 56 (64%) |
| Single | 118 (33%) | 65 (39%) | 27 (28%) | 10 (29%) | 16 (26%) | 23 (26%) |
| Separated or Divorced | 30 (8.3%) | 15 (8.9%) | 8 (8.4%) | 1 (2.9%) | 6 (9.7%) | 7 (8.0%) |
| Widowed | 5 (1.4%) | 2 (1.2%) | 1 (1.1%) | 0 (0%) | 2 (3.2%) | 2 (2.3%) |
| Annual income |  |  |  |  |  |  |
| Less than $20,000 | 5 (1.4%) | 0 (0%) | 2 (2.1%) | 0 (0%) | 3 (4.8%) | 2 (2.3%) |
| $20,000 to $34,999 | 14 (3.9%) | 7 (4.2%) | 3 (3.2%) | 1 (2.9%) | 3 (4.8%) | 4 (4.5%) |
| $35,000 to $49,999 | 16 (4.5%) | 8 (4.8%) | 3 (3.2%) | 3 (8.6%) | 2 (3.2%) | 5 (5.7%) |
| $50,000 to $74,999 | 61 (17%) | 33 (20%) | 13 (14%) | 6 (17%) | 9 (15%) | 14 (16%) |
| $75,000 to $99,999 | 63 (18%) | 32 (19%) | 12 (13%) | 6 (17%) | 13 (21%) | 16 (18%) |
| More than $100,000 | 200 (56%) | 87 (52%) | 62 (65%) | 19 (54%) | 32 (52%) | 47 (53%) |
| House type |  |  |  |  |  |  |
| Detached House | 219 (61%) | 92 (55%) | 62 (65%) | 21 (60%) | 44 (71%) | 59 (67%) |
| Condominium/Apartment | 130 (36%) | 69 (41%) | 29 (31%) | 14 (40%) | 18 (29%) | 29 (33%) |
| Other | 11 (3.1%) | 7 (4.2%) | 4 (4.2%) | 0 (0%) | 0 (0%) |  |
| N household members over the age of 18 |  |  |  |  |  |  |
| 1 | 16 (5.5%) | 11 (8.6%) | 4 (4.9%) | 0 (0%) | 1 (1.9%) | 1 (1.3%) |
| 2 | 196 (67%) | 83 (65%) | 57 (70%) | 23 (79%) | 33 (62%) | 51 (68%) |
| 3 | 55 (19%) | 25 (20%) | 14 (17%) | 5 (17%) | 11 (21%) | 15 (20%) |
| 4 | 15 (5.2%) | 7 (5.5%) | 3 (3.7%) | 0 (0%) | 5 (9.4%) | 4 (5.3%) |
| 5 | 8 (2.7%) | 2 (1.6%) | 3 (3.7%) | 1 (3.4%) | 2 (3.8%) | 3 (4.0%) |
| 6 or more | 1 (0.3%) | 0 (0%) | 0 (0%) | 0 (0%) | 1 (1.9%) | 1 (1.3%) |
| State |  |  |  |  |  |  |
| Connecticut | 357 (99%) | 167 (99%) | 95 (100%) | 34 (97%) | 61 (98%) | 86 (98%) |
| New York | 2 (0.6%) | 1 (0.6%) | 0 (0%) | 1 (2.9%) | 0 (0%) | 1 (1.1%) |
| Massachusetts | 1 (0.3%) | 0 (0%) | 0 (0%) | 0 (0%) | 1 (1.6%) | 1 (1.1%) |
| Flu vaccine |  |  |  |  |  |  |
| Yes, every year | 345 (96%) | 158 (95%) | 93 (98%) | 35 (100%) | 59 (95%) | 85 (97%) |
| Yes, but not every year | 11 (3.1%) | 7 (4.2%) | 1 (1.1%) | 0 (0%) | 3 (4.8%) | 3 (3.4%) |
| No, never | 2 (0.6%) | 1 (0.6%) | 1 (1.1%) | 0 (0%) | 0 (0%) | 0 (0%) |
| Ever tele-worked | 108 (30%) | 43 (26%) | 25 (26%) | 18 (51%) | 22 (35%) | 36 (41%) |
| ^1^Median (IQR); n (%) |  |  |  |  |  |  |

# Supplemental Table 7: Health care personnel contacts over time

|  | **Overall**, N = 469^1^ | **2021-01**, N = 42^1^ | **2021-02**, N = 42^1^ | **2021-03**, N = 21^1^ | **2021-04**, N = 44^1^ | **2021-05**, N = 39^1^ | **2021-08**, N = 14^1^ | **2021-09**, N = 25^1^ | **2021-10**, N = 10^1^ | **2021-11**, N = 14^1^ | **2021-12**, N = 16^1^ | **2022-01**, N = 6^1^ | **2022-02**, N = 6^1^ | **2022-03**, N = 7^1^ | **2022-04**, N = 11^1^ | **2022-05**, N = 100^1^ | **2022-06**, N = 72^1^ |
| --- | --- | --- | --- | --- | --- | --- | --- | --- | --- | --- | --- | --- | --- | --- | --- | --- | --- |
| Finished |  |  |  |  |  |  |  |  |  |  |  |  |  |  |  |  |  |
| FALSE | 52 (11%) | 9 (21%) | 4 (9.5%) | 2 (9.5%) | 6 (14%) | 2 (5.1%) | 3 (21%) | 1 (4.0%) | 0 (0%) | 1 (7.1%) | 0 (0%) | 0 (0%) | 0 (0%) | 0 (0%) | 0 (0%) | 10 (10%) | 14 (19%) |
| TRUE | 417 (89%) | 33 (79%) | 38 (90%) | 19 (90%) | 38 (86%) | 37 (95%) | 11 (79%) | 24 (96%) | 10 (100%) | 13 (93%) | 16 (100%) | 6 (100%) | 6 (100%) | 7 (100%) | 11 (100%) | 90 (90%) | 58 (81%) |
| Building |  |  |  |  |  |  |  |  |  |  |  |  |  |  |  |  |  |
| Hospital1 | 19 (4.2%) | 3 (7.1%) | 3 (7.1%) | 1 (4.8%) | 4 (9.1%) | 2 (5.1%) | 0 (0%) | 0 (0%) | 0 (0%) | 0 (0%) | 0 (0%) | 0 (0%) | 0 (0%) | 0 (0%) | 1 (9.1%) | 5 (5.2%) | 0 (0%) |
| Tower 1 | 91 (20%) | 5 (12%) | 9 (21%) | 5 (24%) | 12 (27%) | 11 (28%) | 4 (36%) | 7 (29%) | 3 (30%) | 3 (23%) | 4 (25%) | 1 (17%) | 1 (17%) | 2 (29%) | 3 (27%) | 11 (11%) | 10 (16%) |
| Tower 2 | 60 (13%) | 7 (17%) | 4 (9.5%) | 1 (4.8%) | 5 (11%) | 7 (18%) | 3 (27%) | 2 (8.3%) | 1 (10%) | 2 (15%) | 1 (6.3%) | 1 (17%) | 1 (17%) | 1 (14%) | 2 (18%) | 13 (13%) | 9 (15%) |
| Tower 3 | 59 (13%) | 11 (26%) | 9 (21%) | 3 (14%) | 10 (23%) | 6 (15%) | 1 (9.1%) | 4 (17%) | 1 (10%) | 3 (23%) | 2 (13%) | 0 (0%) | 0 (0%) | 0 (0%) | 0 (0%) | 8 (8.2%) | 1 (1.6%) |
| Tower 4 | 3aass9 (8.6%) | 5 (12%) | 6 (14%) | 5 (24%) | 5 (11%) | 2 (5.1%) | 0 (0%) | 0 (0%) | 0 (0%) | 0 (0%) | 1 (6.3%) | 0 (0%) | 1 (17%) | 0 (0%) | 0 (0%) | 9 (9.3%) | 5 (8.1%) |
| ED | 45 (10.0%) | 0 (0%) | 1 (2.4%) | 0 (0%) | 0 (0%) | 1 (2.6%) | 0 (0%) | 7 (29%) | 4 (40%) | 4 (31%) | 5 (31%) | 2 (33%) | 2 (33%) | 2 (29%) | 2 (18%) | 11 (11%) | 4 (6.5%) |
| Lab | 22 (4.9%) | 0 (0%) | 0 (0%) | 0 (0%) | 0 (0%) | 0 (0%) | 0 (0%) | 0 (0%) | 0 (0%) | 0 (0%) | 0 (0%) | 0 (0%) | 0 (0%) | 0 (0%) | 0 (0%) | 16 (16%) | 6 (9.7%) |
| Hospital 2 | 31 (6.9%) | 4 (9.5%) | 5 (12%) | 2 (9.5%) | 1 (2.3%) | 3 (7.7%) | 1 (9.1%) | 0 (0%) | 0 (0%) | 1 (7.7%) | 1 (6.3%) | 2 (33%) | 0 (0%) | 1 (14%) | 1 (9.1%) | 3 (3.1%) | 6 (9.7%) |
| Adjacent clinic | 23 (5.1%) | 0 (0%) | 2 (4.8%) | 0 (0%) | 1 (2.3%) | 2 (5.1%) | 0 (0%) | 0 (0%) | 0 (0%) | 0 (0%) | 0 (0%) | 0 (0%) | 0 (0%) | 0 (0%) | 0 (0%) | 10 (10%) | 8 (13%) |
| Off site | 53 (12%) | 7 (17%) | 3 (7.1%) | 4 (19%) | 5 (11%) | 5 (13%) | 1 (9.1%) | 4 (17%) | 1 (10%) | 0 (0%) | 2 (13%) | 0 (0%) | 1 (17%) | 1 (14%) | 2 (18%) | 10 (10%) | 7 (11%) |
| Multi | 9 (2.0%) | 0 (0%) | 0 (0%) | 0 (0%) | 1 (2.3%) | 0 (0%) | 1 (9.1%) | 0 (0%) | 0 (0%) | 0 (0%) | 0 (0%) | 0 (0%) | 0 (0%) | 0 (0%) | 0 (0%) | 1 (1.0%) | 6 (9.7%) |
| Hospital |  |  |  |  |  |  |  |  |  |  |  |  |  |  |  |  |  |
| Adjacent clinic | 3 (0.7%) | 0 (0%) | 0 (0%) | 0 (0%) | 1 (2.3%) | 0 (0%) | 0 (0%) | 0 (0%) | 0 (0%) | 0 (0%) | 0 (0%) | 0 (0%) | 0 (0%) | 0 (0%) | 0 (0%) | 0 (0%) | 2 (3.0%) |
| Multi | 1 (0.2%) | 0 (0%) | 0 (0%) | 0 (0%) | 0 (0%) | 0 (0%) | 0 (0%) | 0 (0%) | 0 (0%) | 0 (0%) | 0 (0%) | 0 (0%) | 0 (0%) | 0 (0%) | 0 (0%) | 1 (1.0%) | 0 (0%) |
| Off site | 55 (12%) | 7 (17%) | 3 (7.1%) | 4 (19%) | 5 (11%) | 5 (13%) | 1 (9.1%) | 4 (17%) | 1 (10%) | 0 (0%) | 2 (13%) | 0 (0%) | 1 (17%) | 1 (14%) | 2 (18%) | 10 (10%) | 9 (14%) |
| Hospital 2 | 32 (7.0%) | 4 (9.5%) | 4 (9.5%) | 2 (9.5%) | 1 (2.3%) | 2 (5.1%) | 1 (9.1%) | 0 (0%) | 0 (0%) | 1 (7.7%) | 1 (6.3%) | 2 (33%) | 0 (0%) | 1 (14%) | 1 (9.1%) | 3 (3.1%) | 9 (14%) |
| Hospital 1 | 364 (80%) | 31 (74%) | 35 (83%) | 15 (71%) | 37 (84%) | 32 (82%) | 9 (82%) | 20 (83%) | 9 (90%) | 12 (92%) | 13 (81%) | 4 (67%) | 5 (83%) | 5 (71%) | 8 (73%) | 83 (86%) | 46 (70%) |
| In vs. out |  |  |  |  |  |  |  |  |  |  |  |  |  |  |  |  |  |
| ED | 1 (0.2%) | 0 (0%) | 0 (0%) | 0 (0%) | 0 (0%) | 0 (0%) | 0 (0%) | 0 (0%) | 0 (0%) | 0 (0%) | 0 (0%) | 0 (0%) | 0 (0%) | 0 (0%) | 0 (0%) | 1 (1.0%) | 0 (0%) |
| Inpatient | 302 (66%) | 31 (74%) | 33 (79%) | 17 (81%) | 31 (70%) | 25 (64%) | 8 (73%) | 17 (71%) | 8 (80%) | 11 (85%) | 13 (81%) | 5 (83%) | 4 (67%) | 6 (86%) | 9 (82%) | 48 (49%) | 36 (55%) |
| Inpatient, Outpatient | 2 (0.4%) | 1 (2.4%) | 0 (0%) | 0 (0%) | 1 (2.3%) | 0 (0%) | 0 (0%) | 0 (0%) | 0 (0%) | 0 (0%) | 0 (0%) | 0 (0%) | 0 (0%) | 0 (0%) | 0 (0%) | 0 (0%) | 0 (0%) |
| Multi | 1 (0.2%) | 0 (0%) | 0 (0%) | 0 (0%) | 0 (0%) | 0 (0%) | 0 (0%) | 0 (0%) | 0 (0%) | 0 (0%) | 0 (0%) | 0 (0%) | 0 (0%) | 0 (0%) | 0 (0%) | 1 (1.0%) | 0 (0%) |
| Outpatient | 149 (33%) | 10 (24%) | 9 (21%) | 4 (19%) | 12 (27%) | 14 (36%) | 3 (27%) | 7 (29%) | 2 (20%) | 2 (15%) | 3 (19%) | 1 (17%) | 2 (33%) | 1 (14%) | 2 (18%) | 47 (48%) | 30 (45%) |
| Day of week |  |  |  |  |  |  |  |  |  |  |  |  |  |  |  |  |  |
| Monday | 77 (16%) | 7 (17%) | 7 (17%) | 7 (33%) | 10 (23%) | 6 (15%) | 4 (31%) | 5 (21%) | 1 (10%) | 3 (21%) | 2 (13%) | 2 (33%) | 2 (33%) | 1 (14%) | 2 (18%) | 15 (15%) | 3 (4.2%) |
| Tuesday | 98 (21%) | 4 (9.5%) | 5 (12%) | 4 (19%) | 10 (23%) | 5 (13%) | 6 (46%) | 3 (13%) | 0 (0%) | 3 (21%) | 3 (19%) | 0 (0%) | 1 (17%) | 2 (29%) | 1 (9.1%) | 49 (49%) | 2 (2.8%) |
| Wednesday | 91 (19%) | 10 (24%) | 5 (12%) | 0 (0%) | 7 (16%) | 3 (7.7%) | 1 (7.7%) | 6 (25%) | 2 (20%) | 3 (21%) | 5 (31%) | 2 (33%) | 2 (33%) | 1 (14%) | 2 (18%) | 8 (8.0%) | 34 (47%) |
| Thursday | 82 (18%) | 8 (19%) | 12 (29%) | 2 (9.5%) | 7 (16%) | 8 (21%) | 0 (0%) | 6 (25%) | 1 (10%) | 2 (14%) | 2 (13%) | 0 (0%) | 0 (0%) | 0 (0%) | 1 (9.1%) | 5 (5.0%) | 28 (39%) |
| Friday | 65 (14%) | 10 (24%) | 7 (17%) | 3 (14%) | 3 (6.8%) | 8 (21%) | 1 (7.7%) | 1 (4.2%) | 5 (50%) | 1 (7.1%) | 2 (13%) | 1 (17%) | 1 (17%) | 3 (43%) | 3 (27%) | 12 (12%) | 4 (5.6%) |
| Saturday | 24 (5.1%) | 2 (4.8%) | 3 (7.1%) | 3 (14%) | 4 (9.1%) | 3 (7.7%) | 1 (7.7%) | 1 (4.2%) | 0 (0%) | 1 (7.1%) | 0 (0%) | 1 (17%) | 0 (0%) | 0 (0%) | 1 (9.1%) | 4 (4.0%) | 0 (0%) |
| Sunday | 30 (6.4%) | 1 (2.4%) | 3 (7.1%) | 2 (9.5%) | 3 (6.8%) | 6 (15%) | 0 (0%) | 2 (8.3%) | 1 (10%) | 1 (7.1%) | 2 (13%) | 0 (0%) | 0 (0%) | 0 (0%) | 1 (9.1%) | 7 (7.0%) | 1 (1.4%) |
| COVID symptoms in past month (Yes) | 318 (68%) | 32 (76%) | 34 (81%) | 12 (57%) | 27 (61%) | 26 (67%) | 12 (86%) | 14 (56%) | 6 (60%) | 11 (79%) | 10 (63%) | 5 (83%) | 3 (50%) | 6 (86%) | 8 (73%) | 70 (70%) | 42 (58%) |
| Duration of symptoms |  |  |  |  |  |  |  |  |  |  |  |  |  |  |  |  |  |
| Few hours | 38 (21%) | 8 (31%) | 6 (30%) | 1 (13%) | 3 (25%) | 1 (13%) | 0 (0%) | 1 (14%) | 1 (25%) | 1 (14%) | 1 (17%) | 0 (0%) | 0 (0%) | 0 (0%) | 0 (0%) | 10 (22%) | 5 (25%) |
| 1 day | 35 (20%) | 2 (7.7%) | 5 (25%) | 1 (13%) | 5 (42%) | 2 (25%) | 1 (33%) | 3 (43%) | 0 (0%) | 3 (43%) | 1 (17%) | 0 (0%) | 0 (0%) | 2 (67%) | 0 (0%) | 7 (15%) | 3 (15%) |
| 2 to 7 days | 60 (34%) | 12 (46%) | 4 (20%) | 3 (38%) | 0 (0%) | 4 (50%) | 2 (67%) | 1 (14%) | 0 (0%) | 3 (43%) | 3 (50%) | 3 (100%) | 0 (0%) | 0 (0%) | 2 (67%) | 17 (37%) | 6 (30%) |
| 1 to 2 weeks | 10 (5.6%) | 0 (0%) | 0 (0%) | 0 (0%) | 1 (8.3%) | 0 (0%) | 0 (0%) | 0 (0%) | 0 (0%) | 0 (0%) | 1 (17%) | 0 (0%) | 0 (0%) | 0 (0%) | 0 (0%) | 4 (8.7%) | 4 (20%) |
| More than 2 weeks | 13 (7.3%) | 0 (0%) | 1 (5.0%) | 1 (13%) | 0 (0%) | 1 (13%) | 0 (0%) | 1 (14%) | 1 (25%) | 0 (0%) | 0 (0%) | 0 (0%) | 1 (50%) | 1 (33%) | 1 (33%) | 4 (8.7%) | 1 (5.0%) |
| None of these | 22 (12%) | 4 (15%) | 4 (20%) | 2 (25%) | 3 (25%) | 0 (0%) | 0 (0%) | 1 (14%) | 2 (50%) | 0 (0%) | 0 (0%) | 0 (0%) | 1 (50%) | 0 (0%) | 0 (0%) | 4 (8.7%) | 1 (5.0%) |
| Work with symptoms (Yes) | 87 (49%) | 4 (15%) | 8 (40%) | 5 (63%) | 5 (42%) | 3 (38%) | 2 (67%) | 5 (71%) | 3 (75%) | 4 (57%) | 3 (50%) | 0 (0%) | 0 (0%) | 2 (67%) | 2 (67%) | 24 (52%) | 17 (85%) |
| Household members with COVID symptoms |  |  |  |  |  |  |  |  |  |  |  |  |  |  |  |  |  |
| Does not apply to me | 30 (10%) | 2 (4.8%) | 4 (9.5%) | 4 (19%) | 5 (12%) | 6 (16%) | 0 (0%) | 1 (14%) | 1 (25%) | 1 (14%) | 2 (33%) | 0 (0%) | 0 (0%) | 0 (0%) | 1 (33%) | 2 (4.3%) | 1 (4.8%) |
| No | 213 (73%) | 32 (76%) | 35 (83%) | 17 (81%) | 33 (80%) | 28 (74%) | 3 (75%) | 6 (86%) | 3 (75%) | 5 (71%) | 2 (33%) | 0 (0%) | 1 (50%) | 2 (67%) | 2 (67%) | 29 (63%) | 15 (71%) |
| Yes | 47 (16%) | 8 (19%) | 3 (7.1%) | 0 (0%) | 3 (7.3%) | 4 (11%) | 1 (25%) | 0 (0%) | 0 (0%) | 1 (14%) | 2 (33%) | 3 (100%) | 1 (50%) | 1 (33%) | 0 (0%) | 15 (33%) | 5 (24%) |
| N shifts on COVID unit |  |  |  |  |  |  |  |  |  |  |  |  |  |  |  |  |  |
| None | 321 (69%) | 30 (71%) | 31 (74%) | 14 (67%) | 30 (75%) | 32 (82%) | 12 (92%) | 15 (63%) | 8 (80%) | 11 (79%) | 10 (63%) | 3 (50%) | 5 (83%) | 6 (86%) | 10 (91%) | 67 (67%) | 37 (52%) |
| 1-5 shifts | 76 (16%) | 6 (14%) | 5 (12%) | 4 (19%) | 8 (20%) | 7 (18%) | 0 (0%) | 2 (8.3%) | 1 (10%) | 2 (14%) | 2 (13%) | 0 (0%) | 0 (0%) | 0 (0%) | 0 (0%) | 19 (19%) | 20 (28%) |
| 6-10 shifts | 30 (6.5%) | 2 (4.8%) | 2 (4.8%) | 3 (14%) | 0 (0%) | 0 (0%) | 1 (7.7%) | 2 (8.3%) | 0 (0%) | 0 (0%) | 0 (0%) | 0 (0%) | 0 (0%) | 0 (0%) | 0 (0%) | 9 (9.0%) | 11 (15%) |
| 11-15 shifts | 22 (4.8%) | 0 (0%) | 2 (4.8%) | 0 (0%) | 1 (2.5%) | 0 (0%) | 0 (0%) | 3 (13%) | 1 (10%) | 1 (7.1%) | 4 (25%) | 2 (33%) | 1 (17%) | 1 (14%) | 1 (9.1%) | 3 (3.0%) | 2 (2.8%) |
| 16-20 shifts | 8 (1.7%) | 4 (9.5%) | 1 (2.4%) | 0 (0%) | 1 (2.5%) | 0 (0%) | 0 (0%) | 2 (8.3%) | 0 (0%) | 0 (0%) | 0 (0%) | 0 (0%) | 0 (0%) | 0 (0%) | 0 (0%) | 0 (0%) | 0 (0%) |
| More than 20 shifts | 3 (0.6%) | 0 (0%) | 1 (2.4%) | 0 (0%) | 0 (0%) | 0 (0%) | 0 (0%) | 0 (0%) | 0 (0%) | 0 (0%) | 0 (0%) | 1 (17%) | 0 (0%) | 0 (0%) | 0 (0%) | 1 (1.0%) | 0 (0%) |
|  | 2 (0.4%) | 0 (0%) | 0 (0%) | 0 (0%) | 0 (0%) | 0 (0%) | 0 (0%) | 0 (0%) | 0 (0%) | 0 (0%) | 0 (0%) | 0 (0%) | 0 (0%) | 0 (0%) | 0 (0%) | 1 (1.0%) | 1 (1.4%) |
| Participated in COVID Code Blue |  |  |  |  |  |  |  |  |  |  |  |  |  |  |  |  |  |
| I don't know | 10 (2.2%) | 0 (0%) | 0 (0%) | 0 (0%) | 1 (2.5%) | 2 (5.1%) | 0 (0%) | 0 (0%) | 0 (0%) | 0 (0%) | 1 (6.3%) | 0 (0%) | 0 (0%) | 0 (0%) | 1 (9.1%) | 2 (2.0%) | 3 (4.3%) |
| No | 425 (93%) | 40 (95%) | 39 (93%) | 20 (95%) | 38 (95%) | 36 (92%) | 12 (92%) | 19 (79%) | 10 (100%) | 13 (93%) | 13 (81%) | 5 (83%) | 6 (100%) | 7 (100%) | 10 (91%) | 94 (95%) | 63 (91%) |
| Yes | 24 (5.2%) | 2 (4.8%) | 3 (7.1%) | 1 (4.8%) | 1 (2.5%) | 1 (2.6%) | 1 (7.7%) | 5 (21%) | 0 (0%) | 1 (7.1%) | 2 (13%) | 1 (17%) | 0 (0%) | 0 (0%) | 0 (0%) | 3 (3.0%) | 3 (4.3%) |
| Within 6 feet of COVID intubation |  |  |  |  |  |  |  |  |  |  |  |  |  |  |  |  |  |
| I don't know | 9 (2.0%) | 0 (0%) | 0 (0%) | 0 (0%) | 0 (0%) | 1 (2.6%) | 0 (0%) | 0 (0%) | 0 (0%) | 0 (0%) | 1 (6.3%) | 1 (17%) | 0 (0%) | 0 (0%) | 1 (9.1%) | 3 (3.0%) | 2 (2.9%) |
| No | 422 (92%) | 40 (95%) | 38 (90%) | 20 (95%) | 39 (98%) | 37 (95%) | 12 (92%) | 18 (75%) | 9 (90%) | 14 (100%) | 13 (81%) | 5 (83%) | 6 (100%) | 7 (100%) | 10 (91%) | 91 (92%) | 63 (91%) |
| Yes | 28 (6.1%) | 2 (4.8%) | 4 (9.5%) | 1 (4.8%) | 1 (2.5%) | 1 (2.6%) | 1 (7.7%) | 6 (25%) | 1 (10%) | 0 (0%) | 2 (13%) | 0 (0%) | 0 (0%) | 0 (0%) | 0 (0%) | 5 (5.1%) | 4 (5.8%) |
| Average N patients direct contact |  |  |  |  |  |  |  |  |  |  |  |  |  |  |  |  |  |
| Less than 5 | 217 (48%) | 22 (54%) | 22 (52%) | 10 (50%) | 20 (51%) | 18 (49%) | 6 (55%) | 11 (46%) | 3 (30%) | 7 (50%) | 9 (56%) | 2 (33%) | 3 (50%) | 3 (43%) | 6 (55%) | 45 (46%) | 30 (45%) |
| 5 to 9 | 131 (29%) | 10 (24%) | 15 (36%) | 7 (35%) | 14 (36%) | 10 (27%) | 2 (18%) | 5 (21%) | 4 (40%) | 3 (21%) | 3 (19%) | 2 (33%) | 1 (17%) | 1 (14%) | 2 (18%) | 34 (35%) | 18 (27%) |
| 10 to 19 | 67 (15%) | 7 (17%) | 3 (7.1%) | 2 (10%) | 2 (5.1%) | 7 (19%) | 2 (18%) | 6 (25%) | 3 (30%) | 4 (29%) | 2 (13%) | 1 (17%) | 1 (17%) | 2 (29%) | 2 (18%) | 13 (13%) | 10 (15%) |
| 20 to 29 | 26 (5.8%) | 2 (4.9%) | 2 (4.8%) | 0 (0%) | 3 (7.7%) | 1 (2.7%) | 1 (9.1%) | 2 (8.3%) | 0 (0%) | 0 (0%) | 2 (13%) | 1 (17%) | 1 (17%) | 1 (14%) | 1 (9.1%) | 5 (5.1%) | 4 (6.1%) |
| 30 to 39 | 2 (0.4%) | 0 (0%) | 0 (0%) | 0 (0%) | 0 (0%) | 0 (0%) | 0 (0%) | 0 (0%) | 0 (0%) | 0 (0%) | 0 (0%) | 0 (0%) | 0 (0%) | 0 (0%) | 0 (0%) | 1 (1.0%) | 1 (1.5%) |
| 40 to 49 | 1 (0.2%) | 0 (0%) | 0 (0%) | 0 (0%) | 0 (0%) | 1 (2.7%) | 0 (0%) | 0 (0%) | 0 (0%) | 0 (0%) | 0 (0%) | 0 (0%) | 0 (0%) | 0 (0%) | 0 (0%) | 0 (0%) | 0 (0%) |
| More than 50 | 4 (0.9%) | 0 (0%) | 0 (0%) | 1 (5.0%) | 0 (0%) | 0 (0%) | 0 (0%) | 0 (0%) | 0 (0%) | 0 (0%) | 0 (0%) | 0 (0%) | 0 (0%) | 0 (0%) | 0 (0%) | 0 (0%) | 3 (4.5%) |
| Average minutes spent with a patient(minutes) |  |  |  |  |  |  |  |  |  |  |  |  |  |  |  |  |  |
| Less than 5 | 144 (32%) | 12 (29%) | 8 (19%) | 5 (25%) | 8 (21%) | 12 (32%) | 4 (36%) | 7 (29%) | 2 (20%) | 4 (29%) | 4 (25%) | 3 (50%) | 2 (33%) | 3 (43%) | 3 (27%) | 43 (44%) | 24 (36%) |
| 5 to 10 | 99 (22%) | 10 (24%) | 11 (26%) | 4 (20%) | 7 (18%) | 8 (22%) | 1 (9.1%) | 5 (21%) | 3 (30%) | 3 (21%) | 5 (31%) | 1 (17%) | 2 (33%) | 1 (14%) | 4 (36%) | 16 (16%) | 18 (27%) |
| 11 to 20 | 89 (20%) | 9 (22%) | 6 (14%) | 2 (10%) | 10 (26%) | 7 (19%) | 5 (45%) | 8 (33%) | 1 (10%) | 3 (21%) | 4 (25%) | 1 (17%) | 0 (0%) | 1 (14%) | 3 (27%) | 16 (16%) | 13 (20%) |
| More than 20 | 116 (26%) | 10 (24%) | 17 (40%) | 9 (45%) | 14 (36%) | 10 (27%) | 1 (9.1%) | 4 (17%) | 4 (40%) | 4 (29%) | 3 (19%) | 1 (17%) | 2 (33%) | 2 (29%) | 1 (9.1%) | 23 (23%) | 11 (17%) |
| Average N patients indirect contact |  |  |  |  |  |  |  |  |  |  |  |  |  |  |  |  |  |
| Less than 5 | 313 (70%) | 32 (78%) | 29 (69%) | 14 (70%) | 31 (79%) | 26 (72%) | 10 (91%) | 15 (63%) | 5 (50%) | 12 (86%) | 9 (56%) | 2 (33%) | 4 (67%) | 5 (71%) | 9 (82%) | 69 (70%) | 41 (62%) |
| 6 to 10 | 70 (16%) | 4 (9.8%) | 9 (21%) | 3 (15%) | 5 (13%) | 4 (11%) | 0 (0%) | 3 (13%) | 0 (0%) | 0 (0%) | 2 (13%) | 4 (67%) | 2 (33%) | 1 (14%) | 1 (9.1%) | 19 (19%) | 13 (20%) |
| 11 to 15 | 31 (6.9%) | 3 (7.3%) | 3 (7.1%) | 1 (5.0%) | 0 (0%) | 5 (14%) | 0 (0%) | 1 (4.2%) | 1 (10%) | 1 (7.1%) | 2 (13%) | 0 (0%) | 0 (0%) | 0 (0%) | 1 (9.1%) | 5 (5.1%) | 8 (12%) |
| 16 to 20 | 19 (4.3%) | 1 (2.4%) | 1 (2.4%) | 1 (5.0%) | 3 (7.7%) | 0 (0%) | 0 (0%) | 4 (17%) | 1 (10%) | 0 (0%) | 1 (6.3%) | 0 (0%) | 0 (0%) | 0 (0%) | 0 (0%) | 4 (4.1%) | 3 (4.5%) |
| 21 to 25 | 6 (1.3%) | 1 (2.4%) | 0 (0%) | 0 (0%) | 0 (0%) | 0 (0%) | 1 (9.1%) | 0 (0%) | 2 (20%) | 0 (0%) | 2 (13%) | 0 (0%) | 0 (0%) | 0 (0%) | 0 (0%) | 0 (0%) | 0 (0%) |
| 26 to 30 | 3 (0.7%) | 0 (0%) | 0 (0%) | 0 (0%) | 0 (0%) | 0 (0%) | 0 (0%) | 1 (4.2%) | 1 (10%) | 1 (7.1%) | 0 (0%) | 0 (0%) | 0 (0%) | 0 (0%) | 0 (0%) | 0 (0%) | 0 (0%) |
| More than 30 | 5 (1.1%) | 0 (0%) | 0 (0%) | 1 (5.0%) | 0 (0%) | 1 (2.8%) | 0 (0%) | 0 (0%) | 0 (0%) | 0 (0%) | 0 (0%) | 0 (0%) | 0 (0%) | 1 (14%) | 0 (0%) | 1 (1.0%) | 1 (1.5%) |
| Average N HCP direct contact |  |  |  |  |  |  |  |  |  |  |  |  |  |  |  |  |  |
| Less than 5 | 74 (17%) | 7 (17%) | 11 (26%) | 4 (20%) | 7 (18%) | 9 (28%) | 1 (9.1%) | 2 (8.3%) | 0 (0%) | 4 (29%) | 2 (13%) | 0 (0%) | 1 (17%) | 0 (0%) | 1 (9.1%) | 19 (20%) | 6 (9.2%) |
| 6 to 10 | 167 (38%) | 14 (34%) | 16 (38%) | 9 (45%) | 21 (54%) | 9 (28%) | 4 (36%) | 11 (46%) | 5 (50%) | 6 (43%) | 4 (25%) | 1 (17%) | 1 (17%) | 4 (57%) | 5 (45%) | 34 (35%) | 23 (35%) |
| 11 to 15 | 90 (20%) | 7 (17%) | 9 (21%) | 3 (15%) | 4 (10%) | 6 (19%) | 1 (9.1%) | 5 (21%) | 3 (30%) | 0 (0%) | 3 (19%) | 4 (67%) | 3 (50%) | 2 (29%) | 1 (9.1%) | 19 (20%) | 20 (31%) |
| 16 to 20 | 52 (12%) | 4 (9.8%) | 1 (2.4%) | 2 (10%) | 6 (15%) | 4 (13%) | 4 (36%) | 2 (8.3%) | 1 (10%) | 0 (0%) | 3 (19%) | 0 (0%) | 0 (0%) | 0 (0%) | 2 (18%) | 11 (11%) | 12 (18%) |
| 21 to 25 | 20 (4.5%) | 4 (9.8%) | 3 (7.1%) | 0 (0%) | 1 (2.6%) | 3 (9.4%) | 0 (0%) | 1 (4.2%) | 0 (0%) | 2 (14%) | 0 (0%) | 0 (0%) | 0 (0%) | 1 (14%) | 0 (0%) | 4 (4.1%) | 1 (1.5%) |
| 26 to 30 | 13 (2.9%) | 4 (9.8%) | 0 (0%) | 1 (5.0%) | 0 (0%) | 0 (0%) | 0 (0%) | 2 (8.3%) | 0 (0%) | 0 (0%) | 1 (6.3%) | 1 (17%) | 0 (0%) | 0 (0%) | 1 (9.1%) | 2 (2.1%) | 1 (1.5%) |
| More than 30 | 25 (5.7%) | 1 (2.4%) | 2 (4.8%) | 1 (5.0%) | 0 (0%) | 1 (3.1%) | 1 (9.1%) | 1 (4.2%) | 1 (10%) | 2 (14%) | 3 (19%) | 0 (0%) | 1 (17%) | 0 (0%) | 1 (9.1%) | 8 (8.2%) | 2 (3.1%) |
| N hours worked on reported day | 9.00 (8.00, 12.00) | 8.00 (8.00, 11.25) | 10.00 (8.00, 12.00) | 12.00 (8.00, 12.00) | 9.00 (7.00, 12.00) | 9.50 (8.00, 12.00) | 10.00 (8.00, 11.00) | 8.00 (8.00, 10.50) | 10.50 (9.25, 12.75) | 10.00 (8.00, 12.00) | 10.00 (8.75, 12.25) | 10.50 (9.25, 11.75) | 9.00 (8.00, 11.50) | 10.00 (9.00, 11.50) | 9.00 (6.50, 12.50) | 8.00 (8.00, 10.00) | 8.00 (8.00, 9.00) |
| Large groups (Y/N) | 282 (63%) | 16 (40%) | 13 (31%) | 6 (32%) | 17 (44%) | 23 (62%) | 8 (62%) | 20 (83%) | 8 (80%) | 8 (57%) | 11 (69%) | 4 (67%) | 5 (83%) | 6 (86%) | 9 (82%) | 81 (83%) | 47 (68%) |
| N contacts reported | 15 (8, 20) | 12 (4, 20) | 10 (4, 19) | 10 (6, 16) | 15 (6, 19) | 14 (10, 24) | 20 (13, 26) | 14 (10, 22) | 12 (11, 19) | 15 (10, 24) | 13 (10, 21) | 18 (16, 28) | 23 (13, 30) | 15 (10, 25) | 20 (10, 26) | 15 (7, 22) | 15 (10, 20) |
| ^1^n (%); Median (IQR) | | | | | | | | | | | | | | | | | |

# Supplemental Table 8: Detailed contact characteristics from longitudinal contact diaries (January 2021-May 2022) and intensive 2-day contact diary, June 2022

|  | | | | | | | | | | | | | | | | | | |
| --- | --- | --- | --- | --- | --- | --- | --- | --- | --- | --- | --- | --- | --- | --- | --- | --- | --- | --- |
| **Characteristic** | **N Direct Patient contact** | | | | **Average Minutes with a Patient** | | | | **N Indirect Patient contact** | | | | **N Direct HCP contact** | | | | | **Average total contacts** |
|  | **Less than 5**, N = 221*^1^* | **5 to 9**, N = 131*^1^* | **10 to 19**, N = 69*^1^* | **20 or more**, N = 33*^1^* | **Less than 5**, N = 145*^1^* | **5 to 10**, N = 101*^1^* | **11 to 20**, N = 91*^1^* | **More than 20**, N = 117*^1^* | **Less than 5**, N = 318*^1^* | **6 to 10**, N = 71*^1^* | **11 to 15**, N = 31*^1^* | **More than 15**, N = 33*^1^* | **Less than 5**, N = 77*^1^* | **6 to 10**, N = 169*^1^* | **11 to 15**, N = 90*^1^* | **16 to 20**, N = 52*^1^* | **More than 20**, N = 59*^1^* | **N = 520***^2^* |
| Age | 45 (35, 56) | 38 (30, 56) | 37 (33, 51) | 34 (29, 42) | 38 (34, 50) | 37 (30, 49) | 44 (37, 59) | 44 (30, 56) | 43 (32, 56) | 38 (31, 49) | 38 (32, 55) | 38 (32, 66) | 45 (35, 55) | 42 (32, 56) | 37 (30, 52) | 38 (30, 52) | 46 (32, 58) |  |
| Gender |  |  |  |  |  |  |  |  |  |  |  |  |  |  |  |  |  |  |
| Female | 198 (90%) | 103 (79%) | 47 (68%) | 21 (64%) | 116 (80%) | 74 (73%) | 71 (78%) | 108 (92%) | 268 (84%) | 54 (76%) | 24 (77%) | 22 (67%) | 71 (92%) | 132 (78%) | 74 (82%) | 45 (87%) | 42 (71%) | 9.00 (8.00, 12.00) |
| Male | 21 (9.5%) | 27 (21%) | 22 (32%) | 12 (36%) | 29 (20%) | 26 (26%) | 18 (20%) | 9 (7.7%) | 48 (15%) | 17 (24%) | 6 (19%) | 11 (33%) | 5 (6.5%) | 35 (21%) | 16 (18%) | 7 (13%) | 17 (29%) | 9.00 (8.00, 10.00) |
| Prefer not to answer | 2 (0.9%) | 1 (0.8%) | 0 (0%) | 0 (0%) | 0 (0%) | 1 (1.0%) | 2 (2.2%) | 0 (0%) | 2 (0.6%) | 0 (0%) | 1 (3.2%) | 0 (0%) | 1 (1.3%) | 2 (1.2%) | 0 (0%) | 0 (0%) | 0 (0%) | 4.00 (4.00, 7.50) |
| Race |  |  |  |  |  |  |  |  |  |  |  |  |  |  |  |  |  |  |
| White or Caucasian | 174 (79%) | 107 (82%) | 59 (86%) | 25 (76%) | 114 (79%) | 80 (79%) | 79 (87%) | 92 (79%) | 250 (79%) | 61 (86%) | 26 (84%) | 27 (82%) | 60 (78%) | 132 (78%) | 80 (89%) | 44 (85%) | 45 (76%) | 8.00 (8.00, 12.00) |
| Asian | 33 (15%) | 11 (8.4%) | 4 (5.8%) | 1 (3.0%) | 19 (13%) | 5 (5.0%) | 5 (5.5%) | 20 (17%) | 42 (13%) | 4 (5.6%) | 1 (3.2%) | 2 (6.1%) | 9 (12%) | 23 (14%) | 9 (10%) | 3 (5.8%) | 5 (8.5%) | 10.00 (8.00, 12.00) |
| Other | 6 (2.7%) | 7 (5.3%) | 4 (5.8%) | 7 (21%) | 5 (3.4%) | 11 (11%) | 4 (4.4%) | 4 (3.4%) | 13 (4.1%) | 3 (4.2%) | 4 (13%) | 4 (12%) | 5 (6.5%) | 8 (4.7%) | 0 (0%) | 3 (5.8%) | 8 (14%) | 8.00 (8.00, 11.50) |
| Black or African American | 8 (3.6%) | 6 (4.6%) | 2 (2.9%) | 0 (0%) | 7 (4.8%) | 5 (5.0%) | 3 (3.3%) | 1 (0.9%) | 13 (4.1%) | 3 (4.2%) | 0 (0%) | 0 (0%) | 3 (3.9%) | 6 (3.6%) | 1 (1.1%) | 2 (3.8%) | 1 (1.7%) | 8.00 (8.00, 9.00) |
| Hispanic/Latino | 16 (7.2%) | 12 (9.2%) | 3 (4.3%) | 3 (9.1%) | 11 (7.6%) | 7 (6.9%) | 9 (9.9%) | 7 (6.0%) | 17 (5.3%) | 10 (14%) | 1 (3.2%) | 6 (18%) | 8 (10%) | 10 (5.9%) | 7 (7.8%) | 5 (9.6%) | 4 (6.8%) |  |
| No |  |  |  |  |  |  |  |  |  |  |  |  |  |  |  |  |  | 9.00 (8.00, 12.00) |
| Yes |  |  |  |  |  |  |  |  |  |  |  |  |  |  |  |  |  | 8.00 (8.00, 12.00) |
| Education |  |  |  |  |  |  |  |  |  |  |  |  |  |  |  |  |  |  |
| High school graduate or GED | 3 (1.4%) | 1 (0.8%) | 1 (1.4%) | 0 (0%) | 3 (2.1%) | 1 (1.0%) | 1 (1.1%) | 0 (0%) | 4 (1.3%) | 1 (1.4%) | 0 (0%) | 0 (0%) | 2 (2.6%) | 1 (0.6%) | 1 (1.1%) | 1 (1.9%) | 0 (0%) | 8.00 (7.00, 8.50) |
| Technical license | 3 (1.4%) | 3 (2.3%) | 0 (0%) | 0 (0%) | 0 (0%) | 3 (3.0%) | 2 (2.2%) | 1 (0.9%) | 5 (1.6%) | 1 (1.4%) | 0 (0%) | 0 (0%) | 0 (0%) | 5 (3.0%) | 0 (0%) | 1 (1.9%) | 0 (0%) | 12.00 (8.00, 12.00) |
| Some college but no degree | 9 (4.1%) | 5 (3.8%) | 2 (2.9%) | 2 (6.1%) | 11 (7.6%) | 5 (5.0%) | 1 (1.1%) | 1 (0.9%) | 13 (4.1%) | 3 (4.2%) | 1 (3.2%) | 1 (3.0%) | 6 (7.8%) | 3 (1.8%) | 1 (1.1%) | 3 (5.8%) | 3 (5.1%) | 8.00 (8.00, 10.00) |
| Associate's degree | 15 (6.8%) | 13 (9.9%) | 7 (10%) | 4 (12%) | 8 (5.5%) | 7 (6.9%) | 3 (3.3%) | 21 (18%) | 28 (8.8%) | 5 (7.0%) | 1 (3.2%) | 5 (15%) | 6 (7.8%) | 18 (11%) | 9 (10%) | 3 (5.8%) | 3 (5.1%) | 12.00 (8.00, 12.00) |
| Bachelor's degree | 76 (34%) | 35 (27%) | 16 (23%) | 5 (15%) | 32 (22%) | 19 (19%) | 25 (27%) | 56 (48%) | 98 (31%) | 19 (27%) | 7 (23%) | 7 (21%) | 21 (27%) | 45 (27%) | 23 (26%) | 12 (23%) | 27 (46%) | 10.00 (8.00, 13.00) |
| Master's degree | 75 (34%) | 31 (24%) | 15 (22%) | 5 (15%) | 60 (41%) | 23 (23%) | 24 (26%) | 19 (16%) | 92 (29%) | 16 (23%) | 12 (39%) | 6 (18%) | 23 (30%) | 42 (25%) | 27 (30%) | 19 (37%) | 15 (25%) | 9.00 (8.00, 10.00) |
| Doctoral degree | 40 (18%) | 43 (33%) | 28 (41%) | 17 (52%) | 31 (21%) | 43 (43%) | 35 (38%) | 19 (16%) | 78 (25%) | 26 (37%) | 10 (32%) | 14 (42%) | 19 (25%) | 55 (33%) | 29 (32%) | 13 (25%) | 11 (19%) | 8.00 (6.00, 10.00) |
| Country of birth |  |  |  |  |  |  |  |  |  |  |  |  |  |  |  |  |  |  |
| United States of America | 187 (85%) | 108 (82%) | 60 (87%) | 26 (79%) | 127 (88%) | 88 (87%) | 76 (84%) | 90 (77%) | 272 (86%) | 54 (76%) | 26 (84%) | 28 (85%) | 63 (82%) | 140 (83%) | 78 (87%) | 48 (92%) | 45 (76%) | 8.00 (8.00, 11.00) |
| Outside of United States | 34 (15%) | 23 (18%) | 9 (13%) | 7 (21%) | 18 (12%) | 13 (13%) | 15 (16%) | 27 (23%) | 46 (14%) | 17 (24%) | 5 (16%) | 5 (15%) | 14 (18%) | 29 (17%) | 12 (13%) | 4 (7.7%) | 14 (24%) | 10.00 (8.00, 12.00) |
| Marital status |  |  |  |  |  |  |  |  |  |  |  |  |  |  |  |  |  |  |
| Married or domestic partnership | 137 (62%) | 86 (66%) | 49 (71%) | 15 (45%) | 97 (67%) | 70 (69%) | 67 (74%) | 53 (45%) | 197 (62%) | 48 (68%) | 20 (65%) | 22 (67%) | 46 (60%) | 112 (66%) | 55 (61%) | 41 (79%) | 29 (49%) | 8.00 (8.00, 10.00) |
| Single | 39 (18%) | 27 (21%) | 16 (23%) | 17 (52%) | 24 (17%) | 24 (24%) | 13 (14%) | 38 (32%) | 68 (21%) | 13 (18%) | 9 (29%) | 9 (27%) | 10 (13%) | 34 (20%) | 27 (30%) | 9 (17%) | 17 (29%) | 11.50 (8.25, 13.00) |
| Separated or Divorced | 43 (19%) | 15 (11%) | 3 (4.3%) | 0 (0%) | 22 (15%) | 2 (2.0%) | 11 (12%) | 26 (22%) | 49 (15%) | 9 (13%) | 1 (3.2%) | 2 (6.1%) | 18 (23%) | 19 (11%) | 8 (8.9%) | 2 (3.8%) | 13 (22%) | 10.00 (8.00, 12.00) |
| Widowed | 2 (0.9%) | 3 (2.3%) | 1 (1.4%) | 1 (3.0%) | 2 (1.4%) | 5 (5.0%) | 0 (0%) | 0 (0%) | 4 (1.3%) | 1 (1.4%) | 1 (3.2%) | 0 (0%) | 3 (3.9%) | 4 (2.4%) | 0 (0%) | 0 (0%) | 0 (0%) | 8.50 (8.00, 9.00) |
| Annual income |  |  |  |  |  |  |  |  |  |  |  |  |  |  |  |  |  |  |
| Less than $20,000 | 2 (0.9%) | 0 (0%) | 2 (2.9%) | 1 (3.0%) | 2 (1.4%) | 1 (1.0%) | 1 (1.1%) | 1 (0.9%) | 3 (0.9%) | 1 (1.4%) | 0 (0%) | 1 (3.0%) | 1 (1.3%) | 0 (0%) | 2 (2.2%) | 1 (1.9%) | 0 (0%) | 8.00 (5.00, 8.00) |
| $20,000 to $34,999 | 5 (2.3%) | 4 (3.1%) | 1 (1.4%) | 1 (3.0%) | 3 (2.1%) | 2 (2.0%) | 3 (3.3%) | 3 (2.6%) | 7 (2.2%) | 2 (2.8%) | 1 (3.2%) | 1 (3.0%) | 0 (0%) | 6 (3.6%) | 3 (3.3%) | 1 (1.9%) | 1 (1.7%) | 8.00 (8.00, 9.50) |
| $35,000 to $49,999 | 11 (5.0%) | 3 (2.3%) | 2 (2.9%) | 2 (6.1%) | 10 (6.9%) | 5 (5.0%) | 3 (3.3%) | 0 (0%) | 13 (4.1%) | 1 (1.4%) | 1 (3.2%) | 3 (9.1%) | 6 (7.8%) | 5 (3.0%) | 1 (1.1%) | 2 (3.8%) | 3 (5.1%) | 8.00 (8.00, 10.00) |
| $50,000 to $74,999 | 19 (8.6%) | 13 (9.9%) | 9 (13%) | 7 (21%) | 16 (11%) | 15 (15%) | 5 (5.5%) | 12 (10%) | 33 (10%) | 8 (11%) | 5 (16%) | 2 (6.1%) | 12 (16%) | 20 (12%) | 7 (7.8%) | 6 (12%) | 3 (5.1%) | 8.00 (8.00, 12.00) |
| $75,000 to $99,999 | 55 (25%) | 44 (34%) | 11 (16%) | 6 (18%) | 17 (12%) | 19 (19%) | 27 (30%) | 53 (45%) | 87 (27%) | 19 (27%) | 6 (19%) | 4 (12%) | 12 (16%) | 41 (24%) | 25 (28%) | 10 (19%) | 26 (44%) | 12.00 (8.00, 13.00) |
| More than $100,000 | 129 (58%) | 67 (51%) | 44 (64%) | 16 (48%) | 97 (67%) | 59 (58%) | 52 (57%) | 48 (41%) | 175 (55%) | 40 (56%) | 18 (58%) | 22 (67%) | 46 (60%) | 97 (57%) | 52 (58%) | 32 (62%) | 26 (44%) | 8.00 (8.00, 10.00) |
| House type |  |  |  |  |  |  |  |  |  |  |  |  |  |  |  |  |  |  |
| Detached House | 162 (73%) | 85 (65%) | 46 (67%) | 16 (48%) | 99 (68%) | 63 (62%) | 66 (73%) | 81 (69%) | 227 (71%) | 43 (61%) | 20 (65%) | 18 (55%) | 54 (70%) | 123 (73%) | 63 (70%) | 33 (63%) | 30 (51%) | 9.00 (8.00, 12.00) |
| Condominium/Apartment | 57 (26%) | 42 (32%) | 22 (32%) | 17 (52%) | 46 (32%) | 33 (33%) | 24 (26%) | 35 (30%) | 87 (27%) | 25 (35%) | 11 (35%) | 15 (45%) | 22 (29%) | 42 (25%) | 26 (29%) | 18 (35%) | 29 (49%) | 9.00 (8.00, 12.00) |
| Other | 2 (0.9%) | 4 (3.1%) | 1 (1.4%) | 0 (0%) | 0 (0%) | 5 (5.0%) | 1 (1.1%) | 1 (0.9%) | 4 (1.3%) | 3 (4.2%) | 0 (0%) | 0 (0%) | 1 (1.3%) | 4 (2.4%) | 1 (1.1%) | 1 (1.9%) | 0 (0%) | 12.00 (12.00, 12.00) |
| N household members |  |  |  |  |  |  |  |  |  |  |  |  |  |  |  |  |  |  |
| 1 | 20 (9.0%) | 16 (12%) | 12 (17%) | 13 (39%) | 14 (9.7%) | 19 (19%) | 11 (12%) | 17 (15%) | 40 (13%) | 9 (13%) | 8 (26%) | 4 (12%) | 4 (5.2%) | 21 (12%) | 12 (13%) | 6 (12%) | 18 (31%) | 10.00 (8.00, 12.00) |
| 2 | 64 (29%) | 43 (33%) | 18 (26%) | 5 (15%) | 47 (32%) | 26 (26%) | 29 (32%) | 28 (24%) | 82 (26%) | 21 (30%) | 8 (26%) | 19 (58%) | 19 (25%) | 41 (24%) | 35 (39%) | 18 (35%) | 16 (27%) | 9.00 (8.00, 10.00) |
| 3 | 55 (25%) | 30 (23%) | 11 (16%) | 6 (18%) | 23 (16%) | 19 (19%) | 22 (24%) | 38 (32%) | 76 (24%) | 13 (18%) | 7 (23%) | 5 (15%) | 17 (22%) | 44 (26%) | 16 (18%) | 11 (21%) | 12 (20%) | 8.00 (8.00, 12.00) |
| 4 | 46 (21%) | 28 (21%) | 19 (28%) | 9 (27%) | 40 (28%) | 21 (21%) | 18 (20%) | 23 (20%) | 74 (23%) | 21 (30%) | 4 (13%) | 3 (9.1%) | 24 (31%) | 40 (24%) | 19 (21%) | 10 (19%) | 8 (14%) | 8.00 (8.00, 10.00) |
| 5 | 28 (13%) | 12 (9.2%) | 3 (4.3%) | 0 (0%) | 15 (10%) | 12 (12%) | 8 (8.8%) | 8 (6.8%) | 35 (11%) | 4 (5.6%) | 2 (6.5%) | 2 (6.1%) | 10 (13%) | 18 (11%) | 7 (7.8%) | 5 (9.6%) | 2 (3.4%) | 10.00 (8.00, 12.00) |
| 6 or more | 8 (3.6%) | 2 (1.5%) | 6 (8.7%) | 0 (0%) | 6 (4.1%) | 4 (4.0%) | 3 (3.3%) | 3 (2.6%) | 11 (3.5%) | 3 (4.2%) | 2 (6.5%) | 0 (0%) | 3 (3.9%) | 5 (3.0%) | 1 (1.1%) | 2 (3.8%) | 3 (5.1%) | 8.00 (4.00, 10.00) |
| N household members over the age of 18 |  |  |  |  |  |  |  |  |  |  |  |  |  |  |  |  |  |  |
| 1 | 7 (3.5%) | 5 (4.3%) | 0 (0%) | 1 (5.0%) | 2 (1.5%) | 0 (0%) | 7 (8.8%) | 4 (4.0%) | 6 (2.2%) | 5 (8.1%) | 0 (0%) | 2 (6.9%) | 4 (5.5%) | 5 (3.4%) | 2 (2.6%) | 1 (2.2%) | 1 (2.4%) | 8.00 (8.00, 9.00) |
| 2 | 127 (63%) | 77 (67%) | 40 (70%) | 14 (70%) | 103 (79%) | 59 (72%) | 49 (61%) | 47 (47%) | 183 (66%) | 38 (61%) | 15 (65%) | 22 (76%) | 51 (70%) | 94 (64%) | 54 (69%) | 31 (67%) | 24 (59%) | 8.00 (8.00, 10.00) |
| 3 | 49 (24%) | 24 (21%) | 12 (21%) | 5 (25%) | 17 (13%) | 19 (23%) | 20 (25%) | 34 (34%) | 66 (24%) | 12 (19%) | 7 (30%) | 4 (14%) | 14 (19%) | 38 (26%) | 11 (14%) | 12 (26%) | 13 (32%) | 10.00 (8.00, 12.00) |
| 4 | 10 (5.0%) | 6 (5.2%) | 3 (5.3%) | 0 (0%) | 5 (3.8%) | 2 (2.4%) | 3 (3.8%) | 9 (9.0%) | 13 (4.7%) | 6 (9.7%) | 0 (0%) | 0 (0%) | 1 (1.4%) | 7 (4.7%) | 7 (9.0%) | 2 (4.3%) | 1 (2.4%) | 13.00 (8.00, 13.00) |
| 5 | 6 (3.0%) | 3 (2.6%) | 2 (3.5%) | 0 (0%) | 2 (1.5%) | 2 (2.4%) | 1 (1.3%) | 6 (6.0%) | 8 (2.9%) | 1 (1.6%) | 1 (4.3%) | 1 (3.4%) | 3 (4.1%) | 4 (2.7%) | 4 (5.1%) | 0 (0%) | 0 (0%) | 10.00 (8.00, 12.00) |
| 6 or more | 2 (1.0%) | 0 (0%) | 0 (0%) | 0 (0%) | 2 (1.5%) | 0 (0%) | 0 (0%) | 0 (0%) | 2 (0.7%) | 0 (0%) | 0 (0%) | 0 (0%) | 0 (0%) | 0 (0%) | 0 (0%) | 0 (0%) | 2 (4.9%) | 11.00 (10.50, 11.50) |
| Unknown |  |  |  |  |  |  |  |  |  |  |  |  |  |  |  |  |  | 71 |
| Jobs summarized |  |  |  |  |  |  |  |  |  |  |  |  |  |  |  |  |  |  |
| Nurse/Nursing Aid | 110 (50%) | 58 (44%) | 18 (26%) | 6 (18%) | 49 (34%) | 34 (34%) | 36 (40%) | 73 (62%) | 140 (44%) | 26 (37%) | 13 (42%) | 12 (36%) | 25 (32%) | 75 (44%) | 42 (47%) | 18 (35%) | 28 (47%) | 11.00 (8.00, 13.00) |
| Doctor | 28 (13%) | 37 (28%) | 22 (32%) | 15 (45%) | 25 (17%) | 39 (39%) | 26 (29%) | 12 (10%) | 63 (20%) | 20 (28%) | 9 (29%) | 10 (30%) | 15 (19%) | 48 (28%) | 22 (24%) | 8 (15%) | 8 (14%) | 8.00 (6.00, 10.00) |
| Midlevel Provider | 25 (11%) | 7 (5.3%) | 11 (16%) | 2 (6.1%) | 16 (11%) | 12 (12%) | 10 (11%) | 7 (6.0%) | 36 (11%) | 5 (7.0%) | 2 (6.5%) | 2 (6.1%) | 12 (16%) | 10 (5.9%) | 6 (6.7%) | 8 (15%) | 9 (15%) | 9.00 (8.00, 10.00) |
| Other | 19 (8.6%) | 6 (4.6%) | 6 (8.7%) | 4 (12%) | 17 (12%) | 12 (12%) | 2 (2.2%) | 4 (3.4%) | 26 (8.2%) | 5 (7.0%) | 0 (0%) | 4 (12%) | 6 (7.8%) | 13 (7.7%) | 7 (7.8%) | 7 (13%) | 1 (1.7%) | 8.00 (7.75, 8.25) |
| Administrative/Social Work | 18 (8.1%) | 8 (6.1%) | 0 (0%) | 3 (9.1%) | 14 (9.7%) | 2 (2.0%) | 12 (13%) | 1 (0.9%) | 19 (6.0%) | 7 (9.9%) | 1 (3.2%) | 2 (6.1%) | 7 (9.1%) | 11 (6.5%) | 5 (5.6%) | 5 (9.6%) | 0 (0%) | 8.00 (8.00, 9.00) |
| Rehabilitation/Transport/Imaging technologist | 3 (1.4%) | 11 (8.4%) | 10 (14%) | 2 (6.1%) | 4 (2.8%) | 1 (1.0%) | 3 (3.3%) | 18 (15%) | 13 (4.1%) | 7 (9.9%) | 5 (16%) | 1 (3.0%) | 5 (6.5%) | 6 (3.6%) | 6 (6.7%) | 5 (9.6%) | 4 (6.8%) | 8.00 (7.00, 8.00) |
| Laboratory | 18 (8.1%) | 1 (0.8%) | 0 (0%) | 0 (0%) | 18 (12%) | 0 (0%) | 1 (1.1%) | 0 (0%) | 19 (6.0%) | 0 (0%) | 0 (0%) | 0 (0%) | 6 (7.8%) | 3 (1.8%) | 2 (2.2%) | 1 (1.9%) | 7 (12%) | 8.00 (8.00, 10.00) |
| Respiratory Therapist | 0 (0%) | 3 (2.3%) | 2 (2.9%) | 1 (3.0%) | 2 (1.4%) | 1 (1.0%) | 1 (1.1%) | 2 (1.7%) | 2 (0.6%) | 1 (1.4%) | 1 (3.2%) | 2 (6.1%) | 1 (1.3%) | 3 (1.8%) | 0 (0%) | 0 (0%) | 2 (3.4%) | 12.00 (12.00, 12.00) |
| Department |  |  |  |  |  |  |  |  |  |  |  |  |  |  |  |  |  |  |
| Primary Care/Internal Medicine | 83 (38%) | 65 (50%) | 15 (22%) | 5 (15%) | 51 (35%) | 36 (36%) | 35 (38%) | 46 (39%) | 120 (38%) | 28 (39%) | 14 (45%) | 6 (18%) | 27 (35%) | 67 (40%) | 39 (43%) | 16 (31%) | 18 (31%) | 9.00 (8.00, 12.00) |
| Other | 67 (30%) | 27 (21%) | 13 (19%) | 9 (27%) | 36 (25%) | 15 (15%) | 22 (24%) | 43 (37%) | 86 (27%) | 16 (23%) | 3 (9.7%) | 10 (30%) | 24 (31%) | 48 (28%) | 12 (13%) | 13 (25%) | 15 (25%) | 9.00 (8.00, 12.00) |
| Surgery | 23 (10%) | 11 (8.4%) | 12 (17%) | 6 (18%) | 7 (4.8%) | 18 (18%) | 13 (14%) | 14 (12%) | 39 (12%) | 6 (8.5%) | 4 (13%) | 3 (9.1%) | 5 (6.5%) | 21 (12%) | 16 (18%) | 6 (12%) | 3 (5.1%) | 10.00 (8.00, 11.75) |
| Emergency Medicine | 6 (2.7%) | 16 (12%) | 19 (28%) | 10 (30%) | 18 (12%) | 22 (22%) | 11 (12%) | 0 (0%) | 20 (6.3%) | 12 (17%) | 7 (23%) | 12 (36%) | 1 (1.3%) | 19 (11%) | 12 (13%) | 5 (9.6%) | 14 (24%) | 9.00 (8.00, 10.50) |
| Pediatrics | 20 (9.0%) | 6 (4.6%) | 1 (1.4%) | 1 (3.0%) | 9 (6.2%) | 8 (7.9%) | 5 (5.5%) | 6 (5.1%) | 24 (7.5%) | 3 (4.2%) | 0 (0%) | 1 (3.0%) | 9 (12%) | 7 (4.1%) | 5 (5.6%) | 6 (12%) | 1 (1.7%) | 8.00 (5.00, 9.50) |
| Laboratory | 19 (8.6%) | 1 (0.8%) | 0 (0%) | 0 (0%) | 19 (13%) | 0 (0%) | 1 (1.1%) | 0 (0%) | 20 (6.3%) | 0 (0%) | 0 (0%) | 0 (0%) | 6 (7.8%) | 5 (3.0%) | 2 (2.2%) | 0 (0%) | 7 (12%) | 8.00 (8.00, 10.00) |
| Rehab/transport | 2 (0.9%) | 3 (2.3%) | 5 (7.2%) | 0 (0%) | 1 (0.7%) | 1 (1.0%) | 4 (4.4%) | 4 (3.4%) | 5 (1.6%) | 3 (4.2%) | 2 (6.5%) | 0 (0%) | 2 (2.6%) | 1 (0.6%) | 3 (3.3%) | 3 (5.8%) | 0 (0%) | 7.50 (5.25, 8.00) |
| Radiology | 1 (0.5%) | 2 (1.5%) | 4 (5.8%) | 2 (6.1%) | 4 (2.8%) | 1 (1.0%) | 0 (0%) | 4 (3.4%) | 4 (1.3%) | 3 (4.2%) | 1 (3.2%) | 1 (3.0%) | 3 (3.9%) | 1 (0.6%) | 1 (1.1%) | 3 (5.8%) | 1 (1.7%) | 8.00 (8.00, 8.00) |
| Years working as HCP |  |  |  |  |  |  |  |  |  |  |  |  |  |  |  |  |  |  |
| 10 years or less | 72 (33%) | 53 (40%) | 36 (52%) | 22 (67%) | 50 (34%) | 58 (57%) | 30 (33%) | 45 (38%) | 124 (39%) | 31 (44%) | 14 (45%) | 14 (42%) | 25 (32%) | 68 (40%) | 46 (51%) | 22 (42%) | 21 (36%) | 9.00 (8.00, 12.00) |
| 20 years or more | 90 (41%) | 40 (31%) | 20 (29%) | 2 (6.1%) | 42 (29%) | 26 (26%) | 36 (40%) | 48 (41%) | 117 (37%) | 15 (21%) | 8 (26%) | 11 (33%) | 25 (32%) | 59 (35%) | 27 (30%) | 13 (25%) | 25 (42%) | 9.00 (8.00, 12.00) |
| 11 to 20 years | 59 (27%) | 38 (29%) | 13 (19%) | 9 (27%) | 53 (37%) | 17 (17%) | 25 (27%) | 24 (21%) | 77 (24%) | 25 (35%) | 9 (29%) | 8 (24%) | 27 (35%) | 42 (25%) | 17 (19%) | 17 (33%) | 13 (22%) | 8.00 (8.00, 10.00) |
| Average number of hours worked per week |  |  |  |  |  |  |  |  |  |  |  |  |  |  |  |  |  |  |
| 0-20 hours per week | 14 (6.3%) | 2 (1.5%) | 4 (5.8%) | 0 (0%) | 7 (4.8%) | 5 (5.0%) | 2 (2.2%) | 6 (5.1%) | 16 (5.0%) | 2 (2.9%) | 2 (6.5%) | 0 (0%) | 9 (12%) | 9 (5.3%) | 0 (0%) | 1 (1.9%) | 0 (0%) | 6.00 (4.75, 8.00) |
| 21-40 hours per week | 172 (78%) | 95 (73%) | 57 (83%) | 27 (82%) | 107 (74%) | 73 (73%) | 71 (78%) | 100 (85%) | 251 (79%) | 53 (76%) | 20 (65%) | 27 (82%) | 53 (70%) | 128 (76%) | 74 (82%) | 41 (79%) | 50 (85%) | 9.00 (8.00, 12.00) |
| 41-60 hours per week | 34 (15%) | 31 (24%) | 7 (10%) | 6 (18%) | 31 (21%) | 19 (19%) | 18 (20%) | 10 (8.5%) | 48 (15%) | 14 (20%) | 9 (29%) | 6 (18%) | 13 (17%) | 29 (17%) | 16 (18%) | 10 (19%) | 9 (15%) | 9.00 (7.00, 10.00) |
| 60 hours per week or more | 1 (0.5%) | 2 (1.5%) | 1 (1.4%) | 0 (0%) | 0 (0%) | 3 (3.0%) | 0 (0%) | 1 (0.9%) | 3 (0.9%) | 1 (1.4%) | 0 (0%) | 0 (0%) | 1 (1.3%) | 3 (1.8%) | 0 (0%) | 0 (0%) | 0 (0%) | 10.00 (8.00, 12.50) |
| Unknown |  |  |  |  |  |  |  |  |  |  |  |  |  |  |  |  |  | 1 |
| History of COVID-19 at time of enrollment |  |  |  |  |  |  |  |  |  |  |  |  |  |  |  |  |  |  |
| No | 175 (79%) | 106 (81%) | 55 (80%) | 28 (85%) | 126 (87%) | 85 (84%) | 74 (81%) | 79 (68%) | 249 (78%) | 61 (86%) | 27 (87%) | 26 (79%) | 63 (82%) | 136 (80%) | 79 (88%) | 45 (87%) | 37 (63%) | 9.00 (8.00, 11.00) |
| Yes, and it was confirmed by a diagnostic test | 39 (18%) | 23 (18%) | 14 (20%) | 5 (15%) | 16 (11%) | 14 (14%) | 17 (19%) | 34 (29%) | 60 (19%) | 10 (14%) | 4 (13%) | 7 (21%) | 11 (14%) | 30 (18%) | 10 (11%) | 6 (12%) | 22 (37%) | 9.00 (8.00, 12.00) |
| Yes, but it was not confirmed by a diagnostic test | 7 (3.2%) | 2 (1.5%) | 0 (0%) | 0 (0%) | 3 (2.1%) | 2 (2.0%) | 0 (0%) | 4 (3.4%) | 9 (2.8%) | 0 (0%) | 0 (0%) | 0 (0%) | 3 (3.9%) | 3 (1.8%) | 1 (1.1%) | 1 (1.9%) | 0 (0%) | 12.00 (8.75, 13.00) |
| Vaccinated for COVID-19 at time of enrollment | 184 (83%) | 110 (84%) | 62 (90%) | 31 (94%) | 137 (94%) | 88 (87%) | 73 (80%) | 89 (76%) | 269 (85%) | 60 (85%) | 27 (87%) | 30 (91%) | 61 (79%) | 149 (88%) | 80 (89%) | 45 (87%) | 46 (78%) |  |
| No |  |  |  |  |  |  |  |  |  |  |  |  |  |  |  |  |  | 8.00 (7.00, 12.25) |
| Yes |  |  |  |  |  |  |  |  |  |  |  |  |  |  |  |  |  | 9.00 (8.00, 12.00) |
| *^1^* Median (IQR); n (%) | | | | | | | | | | | | | | | | | | |
| *^2^* Q29: Median (IQR) | | | | | | | | | | | | | | | | | | |

# Supplemental Table 9: Detailed contact characteristics from intensive 2-day contact diary, June 2022

| **Characteristic** | **Day 1**, N = 1,420*^1^* | **Day 2**, N = 1,130*^1^* |
| --- | --- | --- |
| Relationship with contact |  |  |
| Colleague | 909 (64%) | 683 (60%) |
| Patient | 301 (21%) | 269 (24%) |
| Do not know this person personally, not a patient | 95 (6.7%) | 62 (5.5%) |
| Household member | 73 (5.1%) | 70 (6.2%) |
| Friend/acquaintance | 19 (1.3%) | 32 (2.8%) |
| Relative | 23 (1.6%) | 14 (1.2%) |
| Contact gender |  |  |
| Female | 949 (67%) | 752 (67%) |
| Male | 459 (32%) | 367 (32%) |
| I don't know | 11 (0.8%) | 10 (0.9%) |
| Non-binary | 1 (<0.1%) | 1 (<0.1%) |
| Contact estimated age |  |  |
| Less than 1 year old | 25 (1.8%) | 18 (1.6%) |
| 1 to 9 years old | 35 (2.5%) | 36 (3.2%) |
| 10 to 19 years old | 24 (1.7%) | 27 (2.4%) |
| 20 to 29 years old | 248 (17%) | 232 (21%) |
| 30 to 39 years old | 368 (26%) | 262 (23%) |
| 40 to 49 years old | 256 (18%) | 193 (17%) |
| 50 to 59 years old | 250 (18%) | 179 (16%) |
| 60 to 69 years old | 131 (9.2%) | 107 (9.5%) |
| 70 to 79 years old | 57 (4.0%) | 49 (4.3%) |
| 80 years and older | 26 (1.8%) | 27 (2.4%) |
| Contact type |  |  |
| Non-physical contact | 949 (67%) | 805 (71%) |
| Physical contact | 257 (18%) | 211 (19%) |
| Direct proximity | 214 (15%) | 114 (10%) |
| Contact duration for 24-hour period |  |  |
| Less than 5 minutes | 237 (17%) | 194 (17%) |
| Between 5 to 15 minutes | 365 (26%) | 275 (24%) |
| Between 15 minutes to 1 hour | 418 (29%) | 358 (32%) |
| Between 1 hour to 4 hours | 220 (15%) | 165 (15%) |
| More than 4 hours | 180 (13%) | 138 (12%) |
| Frequency of contact |  |  |
| Daily or almost daily | 443 (31%) | 352 (31%) |
| I don't have contact with this person generally | 358 (25%) | 295 (26%) |
| About 1 to 2 times per week | 362 (25%) | 289 (26%) |
| About 1 to 2 times per month | 182 (13%) | 134 (12%) |
| Less than once per month | 75 (5.3%) | 59 (5.2%) |
| Unknown | 0 | 1 |
| Was participant wearing a sensor |  |  |
| No | 1,108 (78%) | 866 (77%) |
| Unsure | 186 (13%) | 143 (13%) |
| Yes | 126 (8.9%) | 119 (11%) |
| Unknown | 0 | 2 |
| Contact location |  |  |
| Hospital | 814 (57%) | 595 (53%) |
| Ambulatory | 232 (16%) | 157 (14%) |
| Other (work associated) | 114 (8.0%) | 96 (8.5%) |
| Home | 98 (6.9%) | 95 (8.4%) |
| ER | 91 (6.4%) | 101 (8.9%) |
| Hospital, Ambulatory | 41 (2.9%) | 48 (4.2%) |
| Other (non-work associated) | 13 (0.9%) | 27 (2.4%) |
| Hospital, Other (work associated) | 10 (0.7%) | 2 (0.2%) |
| Hospital, Home | 4 (0.3%) | 1 (<0.1%) |
| Hospital, Other (non-work associated) | 3 (0.2%) | 2 (0.2%) |
| Hospital, ER | 0 (0%) | 4 (0.4%) |
| Ambulatory, Home | 0 (0%) | 2 (0.2%) |
| *^1^* n (%) | | |

# Supplemental Figure 1: Geographic of primary hospital lay out

Medical tower 1 consisted of medical intensive care units (ICUs), oncology medical floors, outpatient infusion therapy, radiology suites and oncology clinics. Medical tower 2 had transplant care, cardiac ICUs, Pediatric ICUs and pediatric floors and maternity care. Medical tower 3 had surgical ICU, cardiac ICU, neuro ICUs, and medical/surgical floors and the emergency room. Medical tower 4 has medical/surgical floors. Each tower also has operative rooms and or post-operative care areas. The laboratory building and adjacent clinics are connected via walkways.


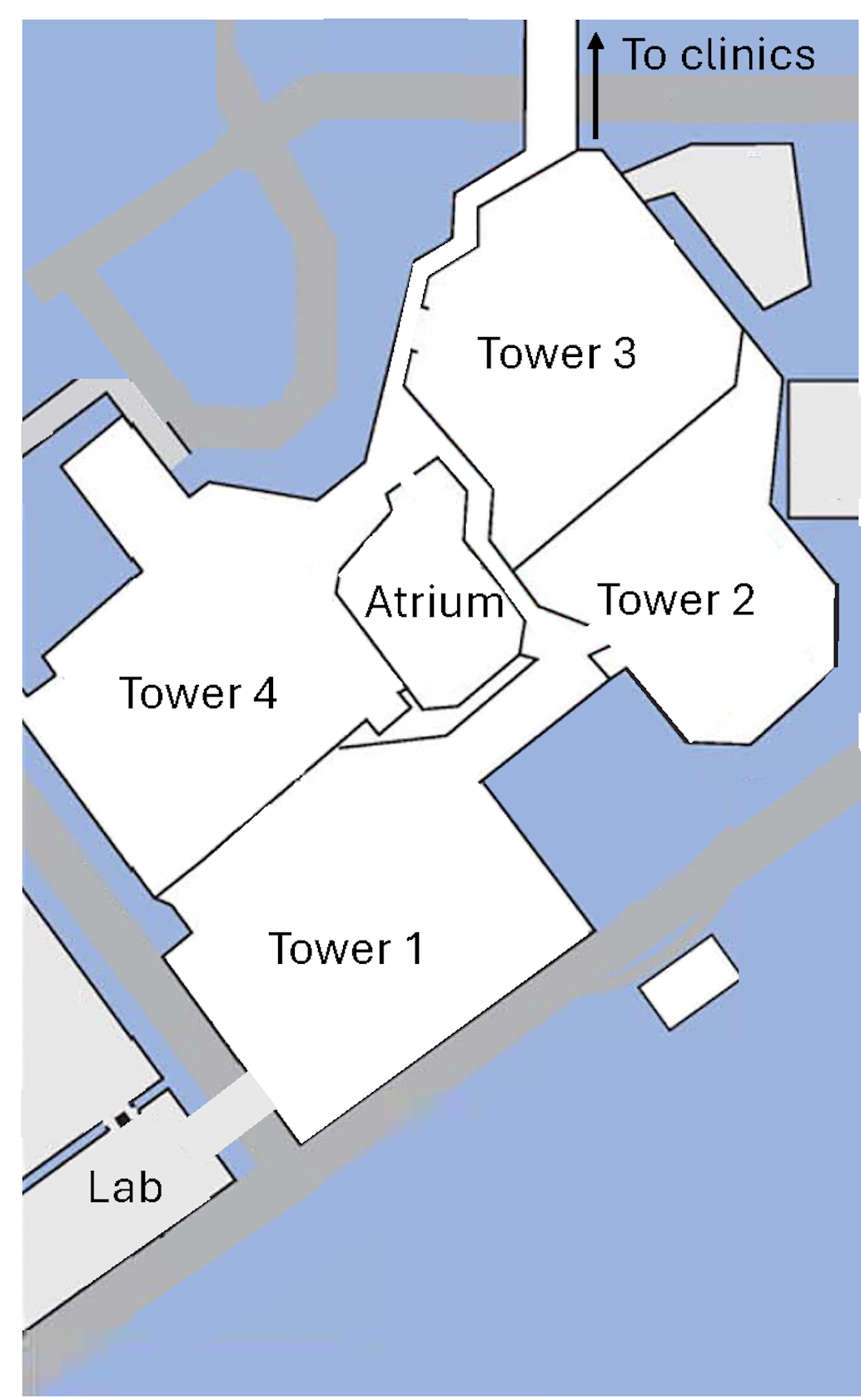


# Supplemental Figure 2: Age-contact matrices by locations and other individuals

Age-contact matrices by locations and other individuals: A: Location of contact at the hospital. B: Location of contact at the home, C: average contacts with household members D: Location of contact at the Emergency Room. E: location of contact other work associated. F: Average contacts with other contacts


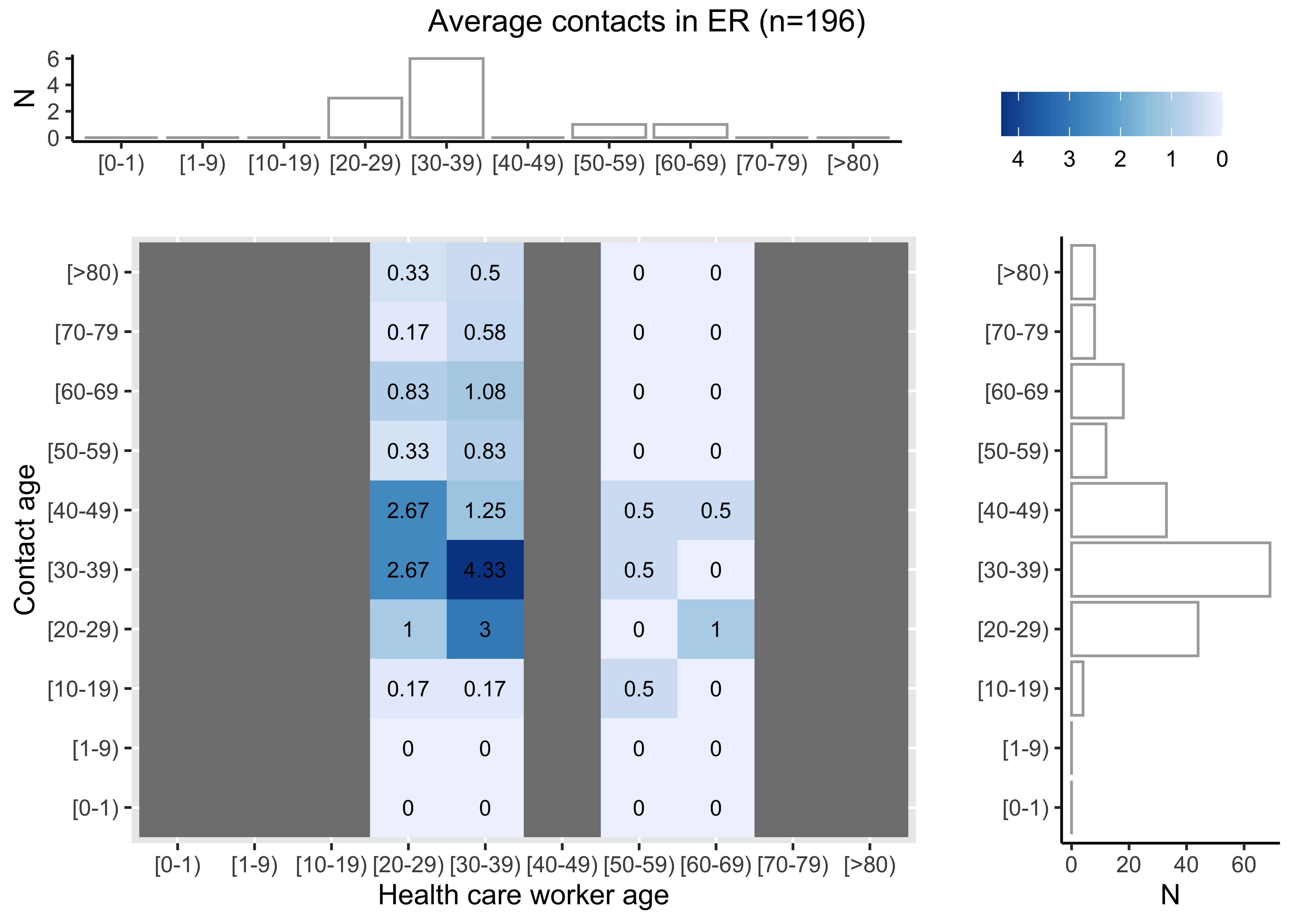

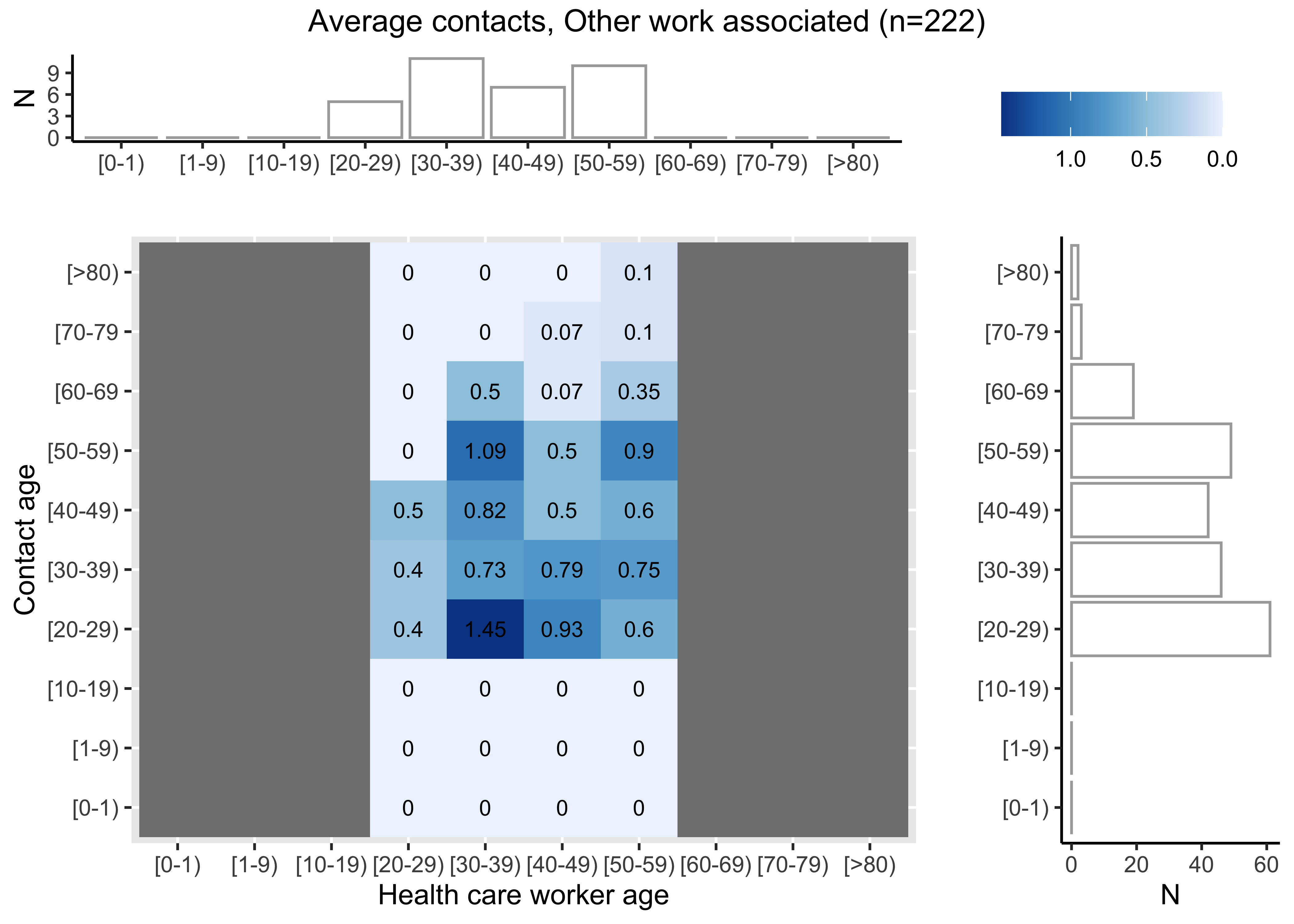

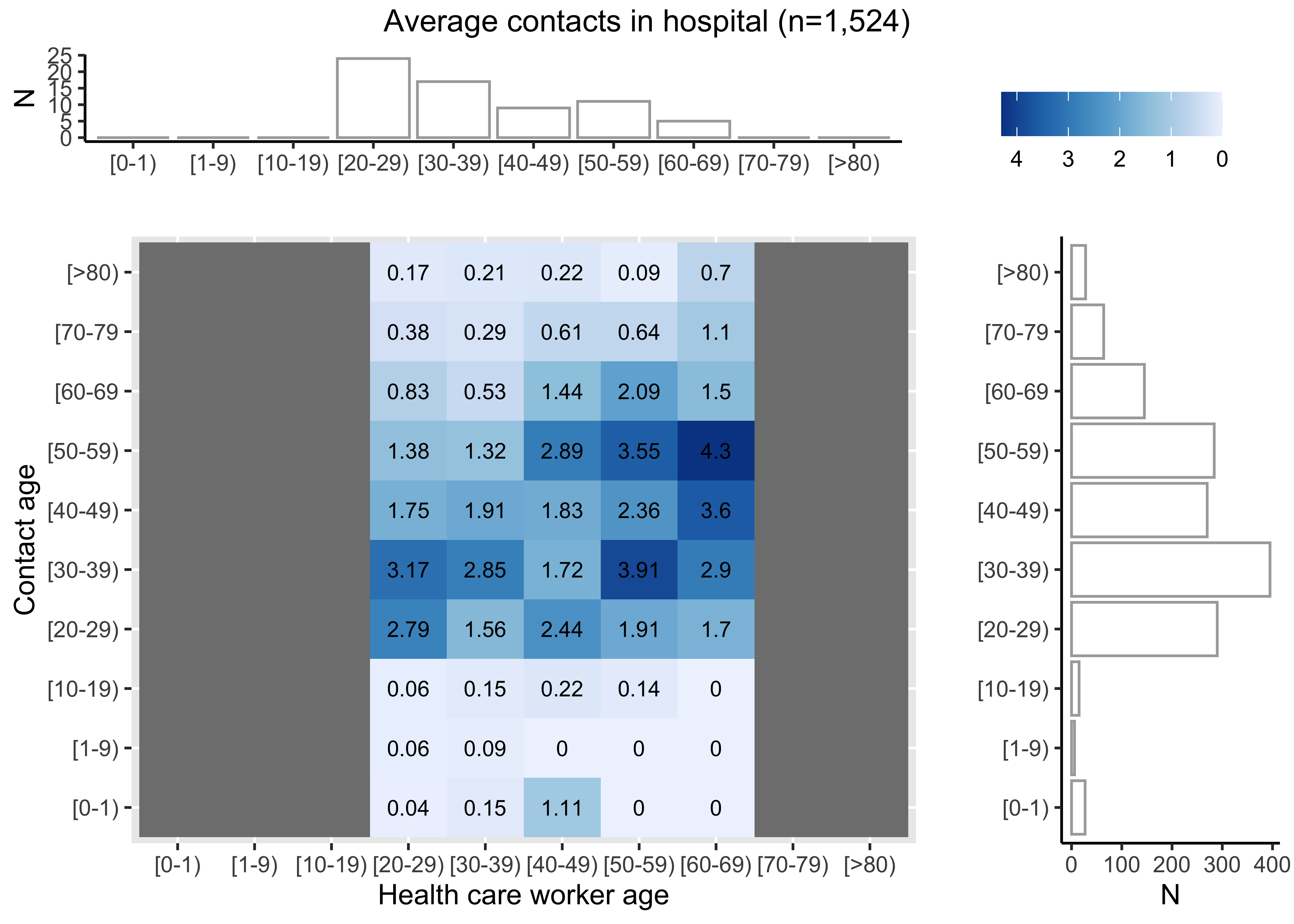

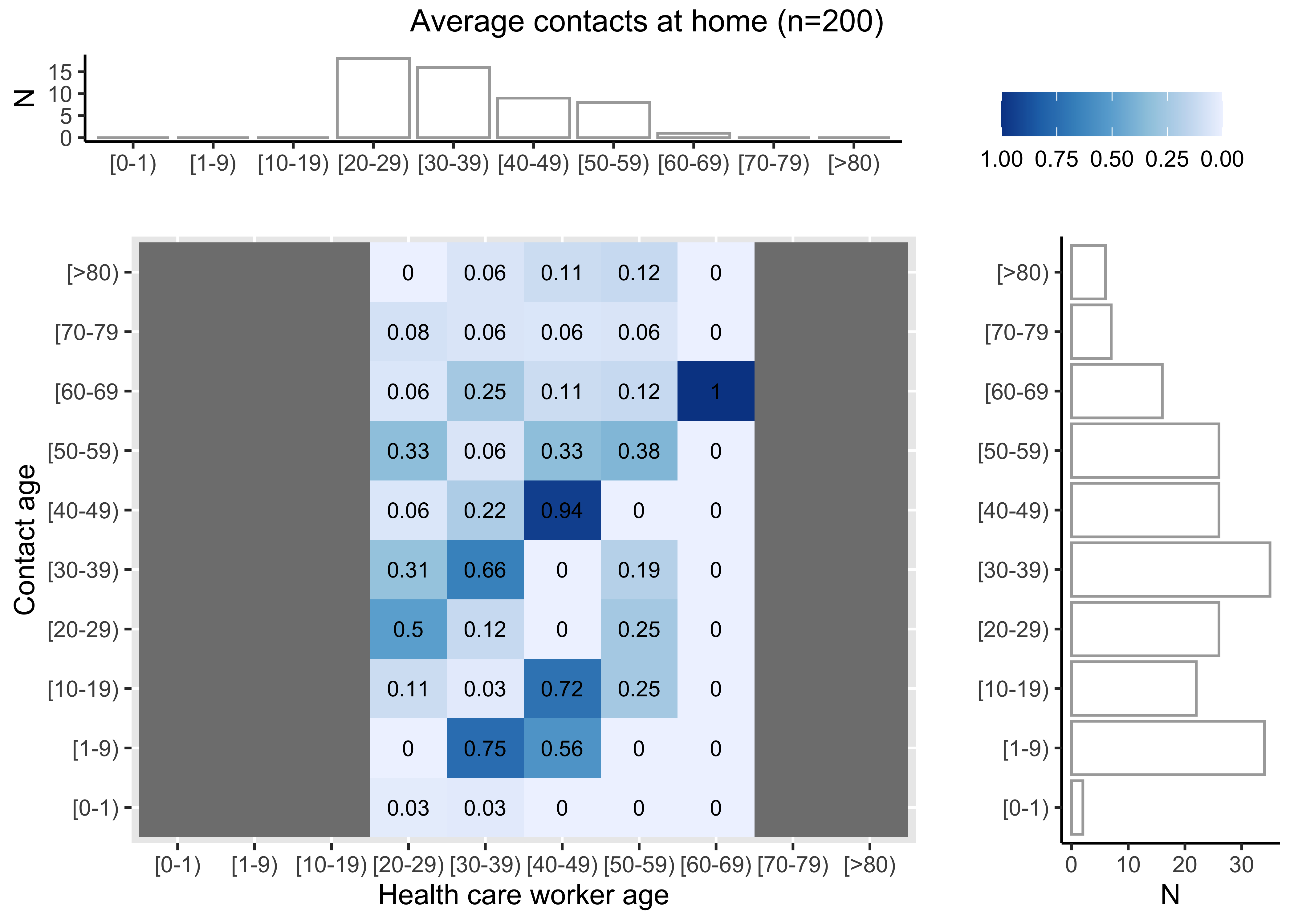

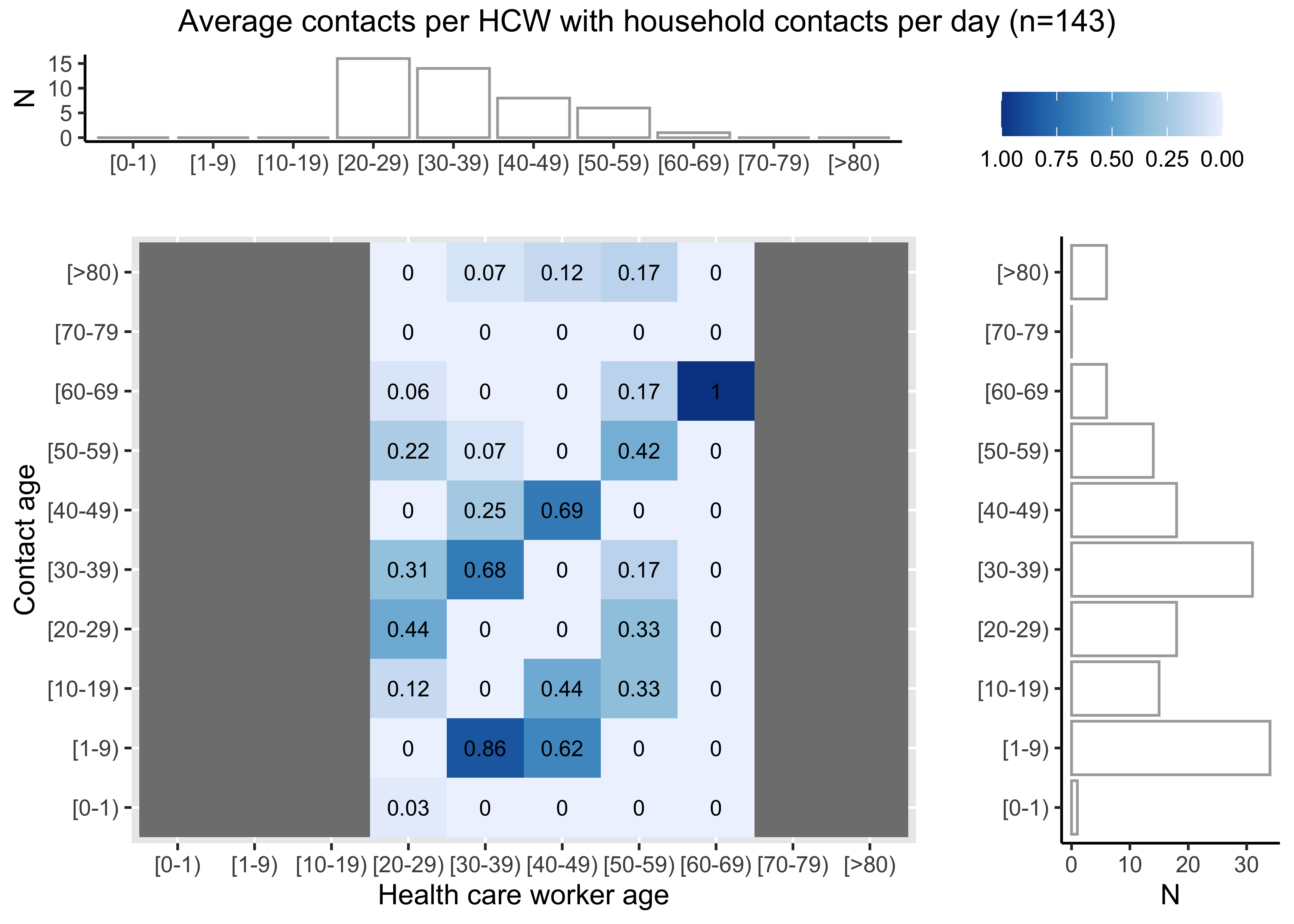

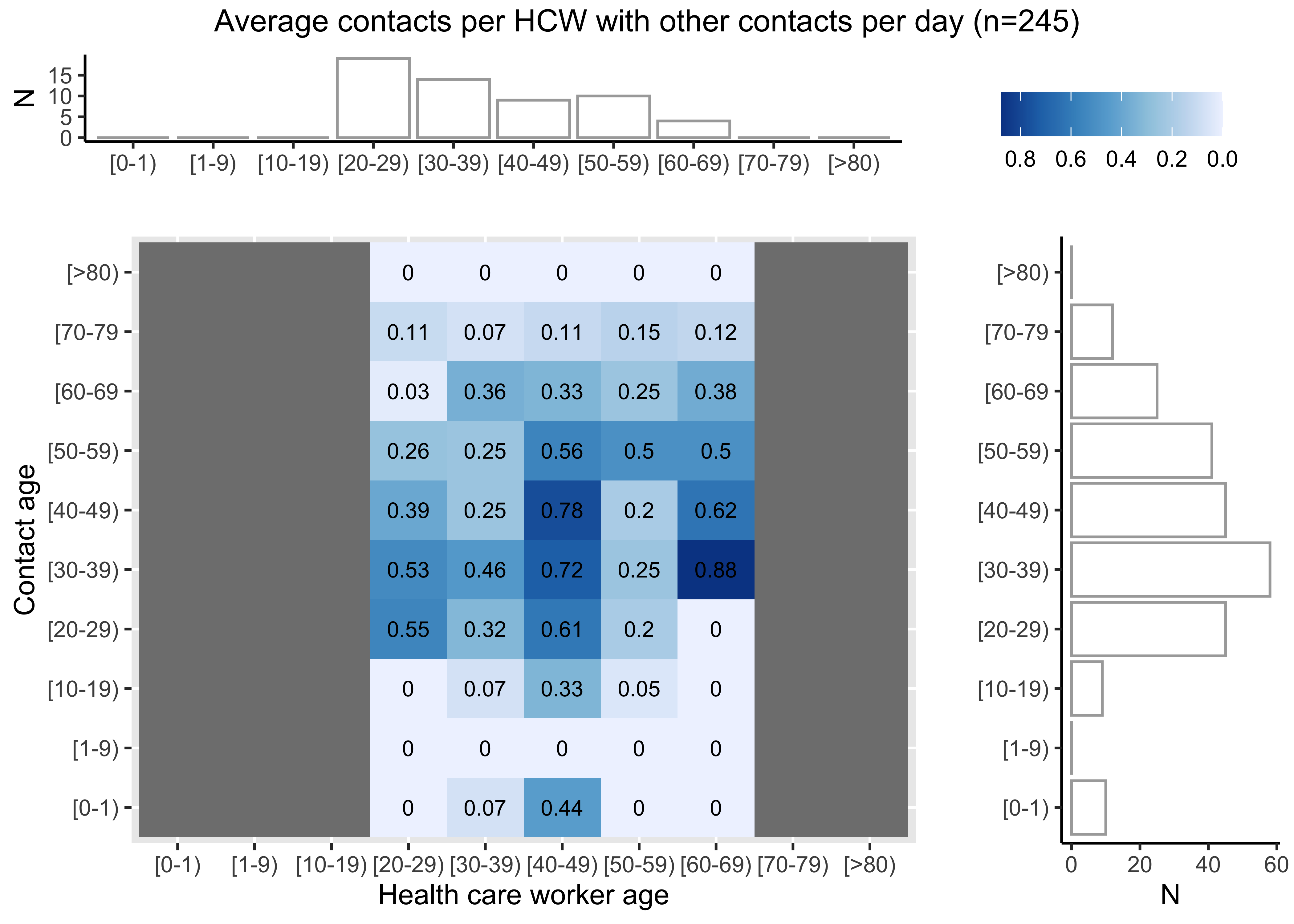


A

B

C

D

E

F

# Supplemental Figure 3: Age-contact matrices by contact relationship (patient vs HCP) and contact type (direct proximity, non-physical, physical contact)

A: Direct proximity with patients. B non-physical contact with patients C: Physical contact with patients. D: direct proximity with colleague. E: non-physical contact with colleagues F: Average physical contact with colleagues


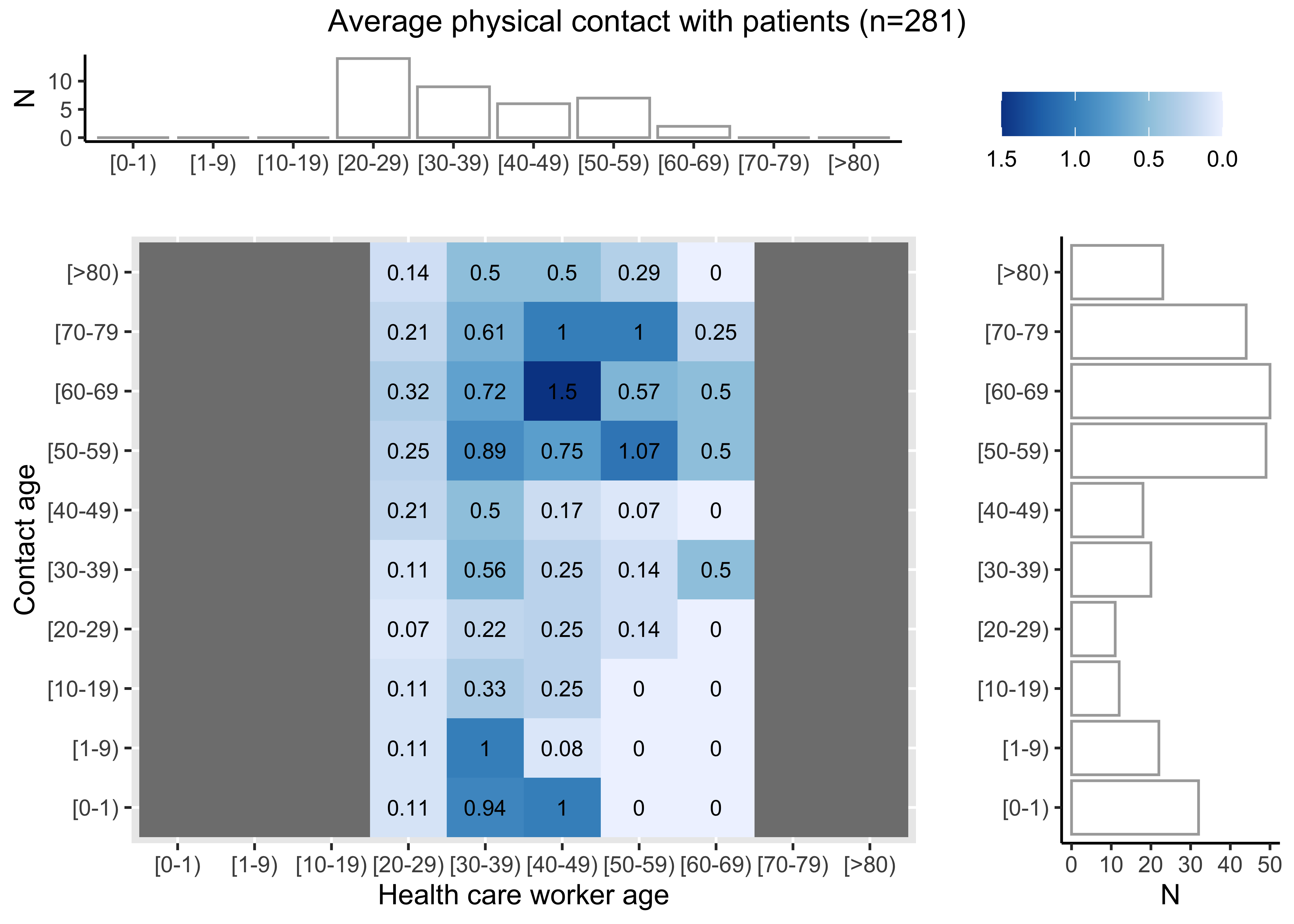

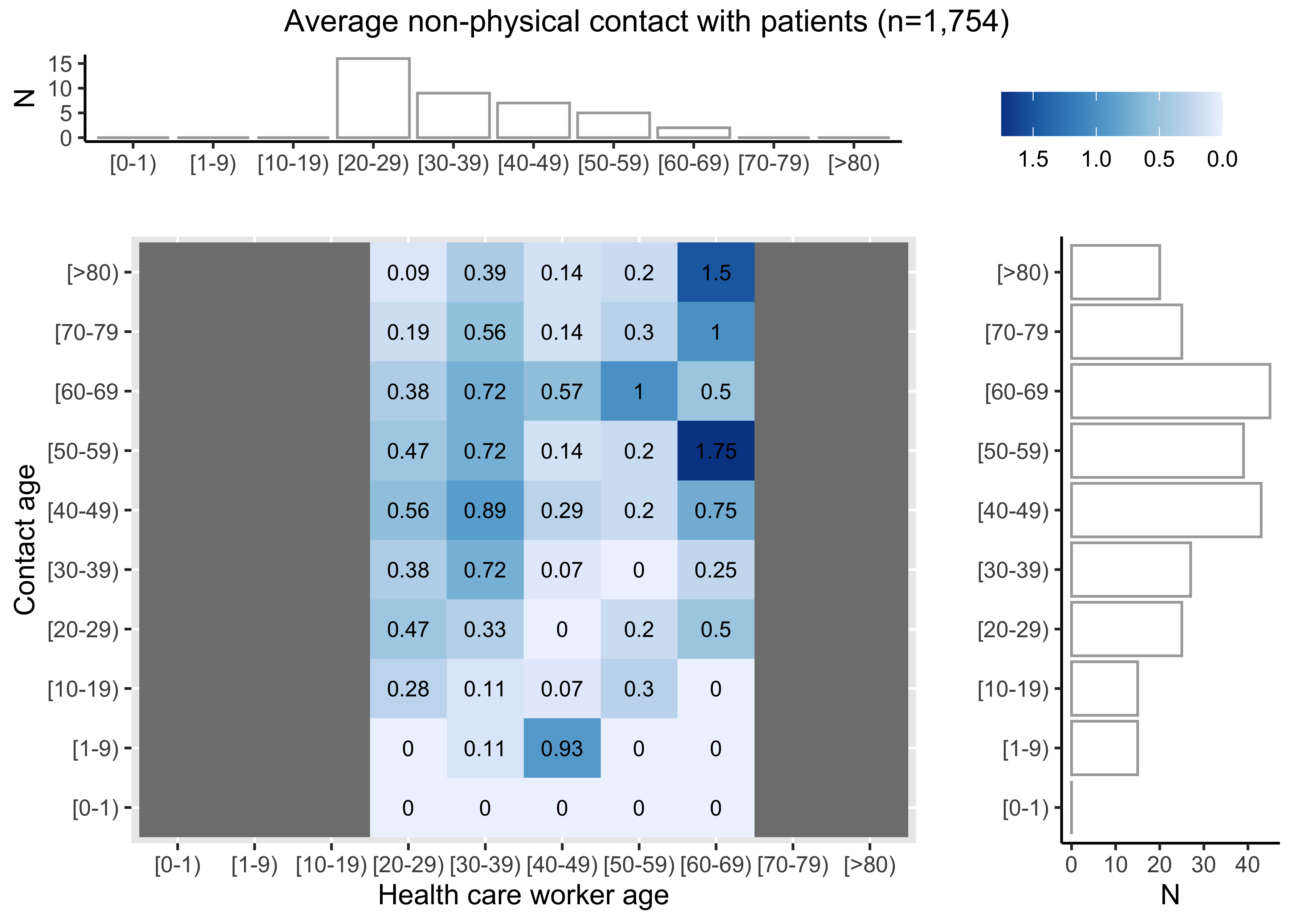

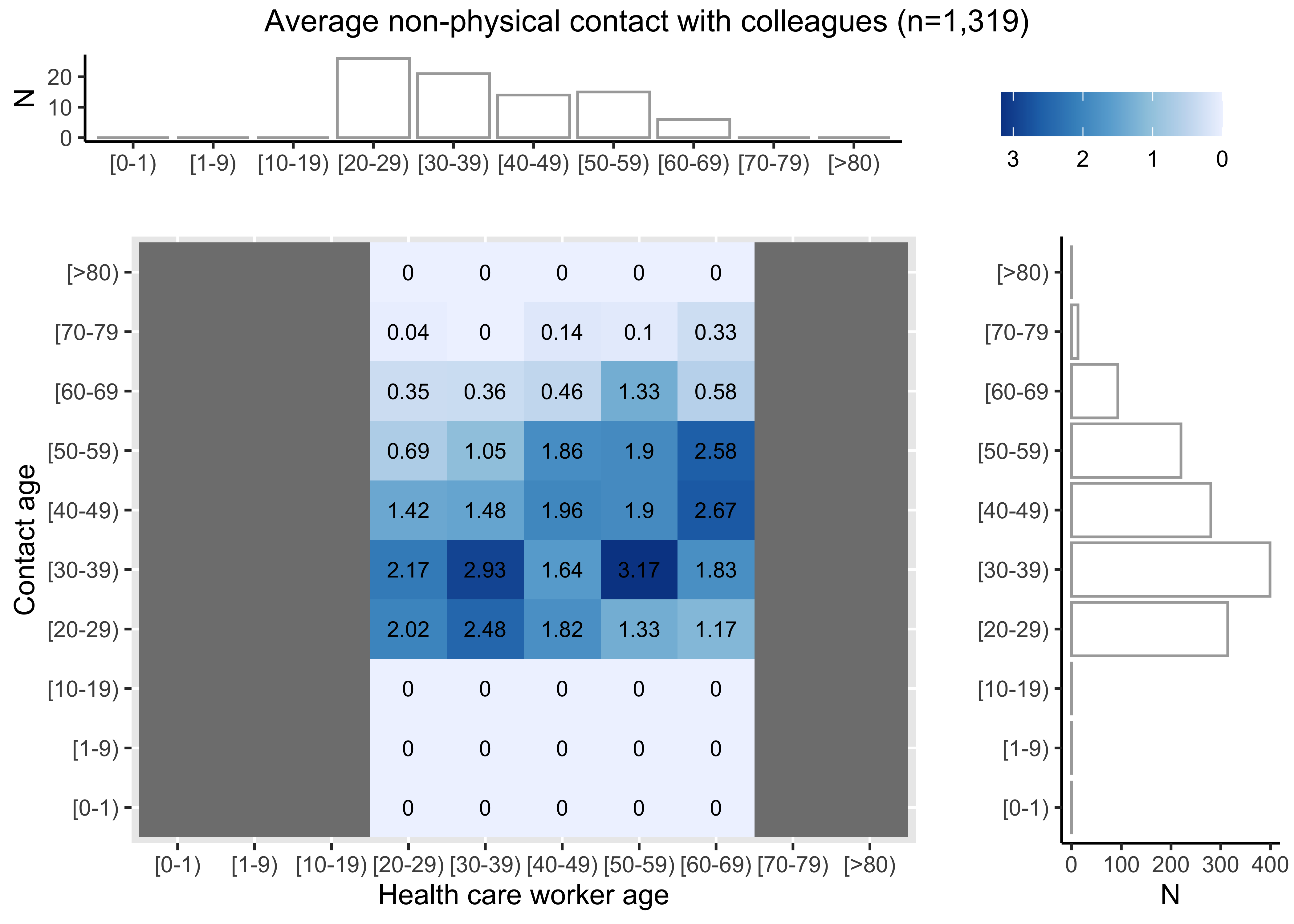

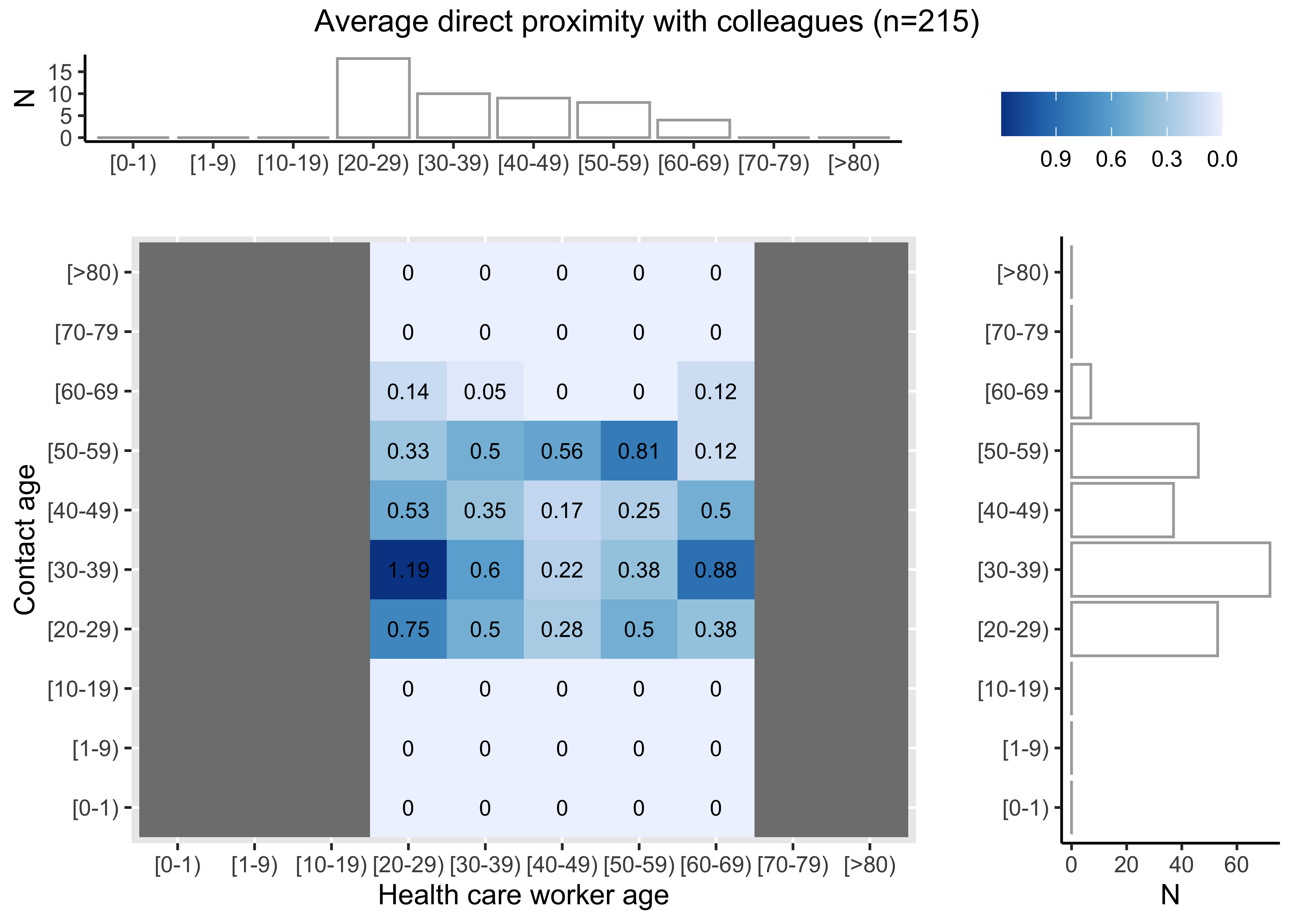

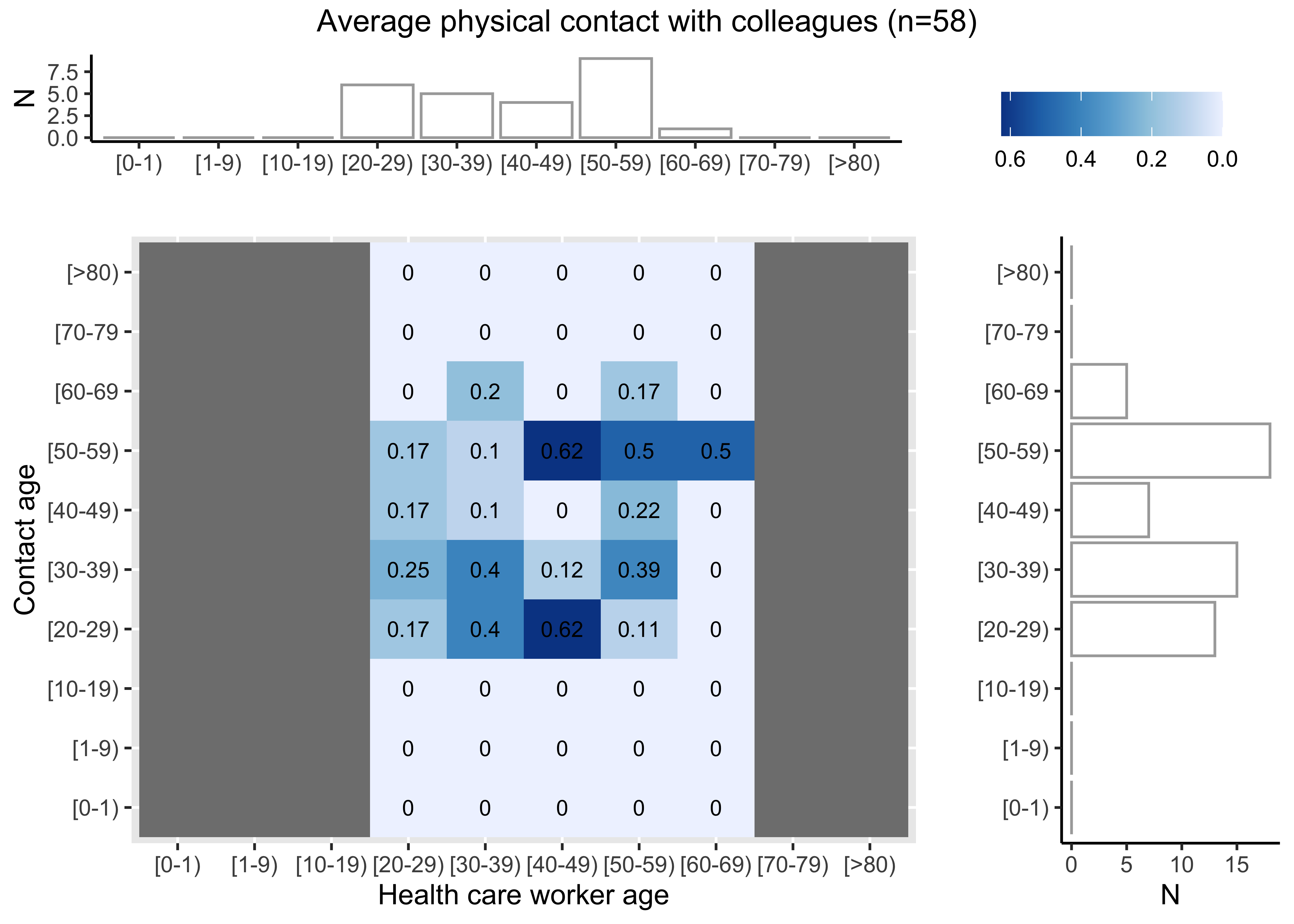

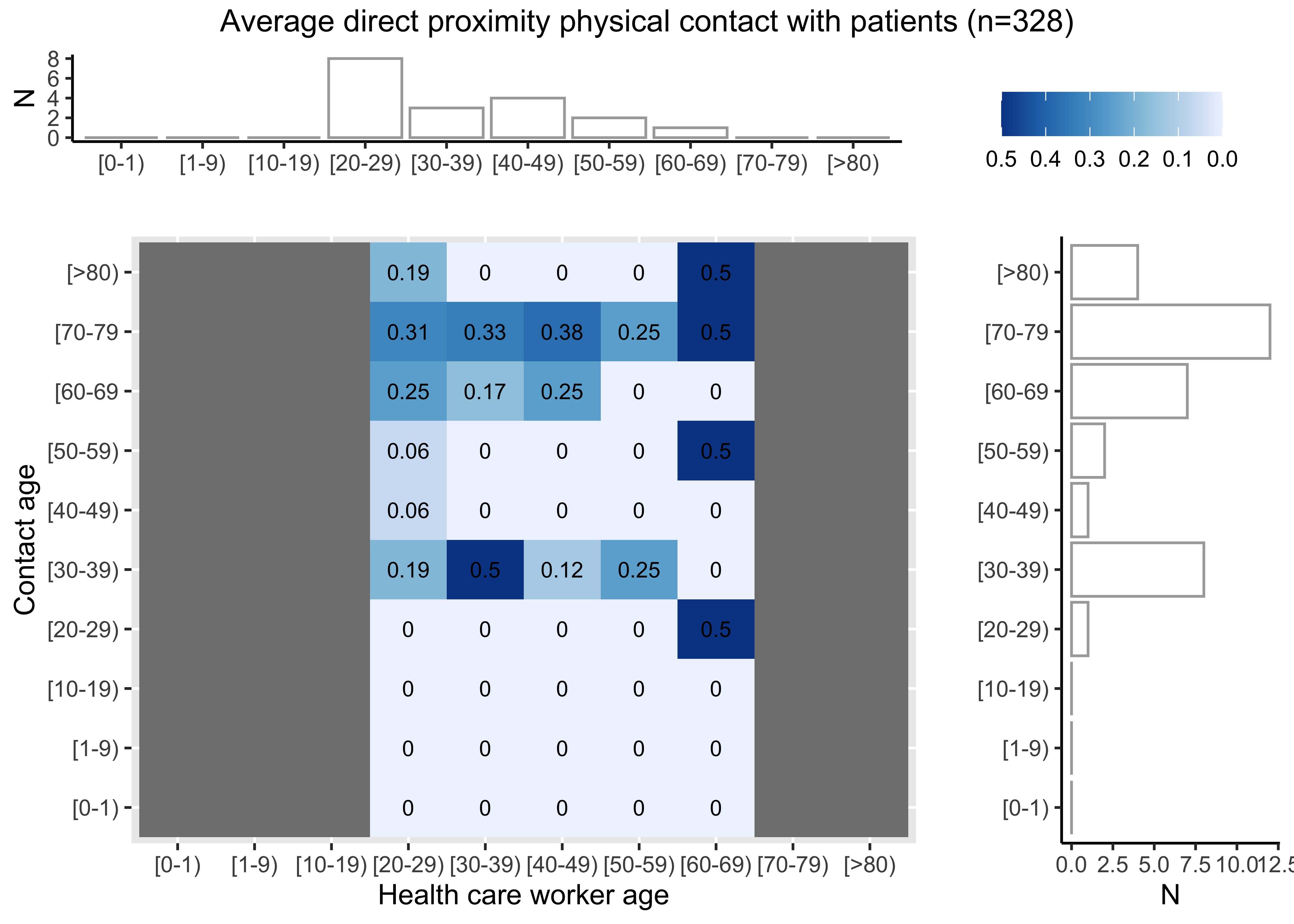


A

B

D

E

F

C

# Supplemental Figure 4: Detailed vs. summative reported contacts in June 2022 intensive contact diary


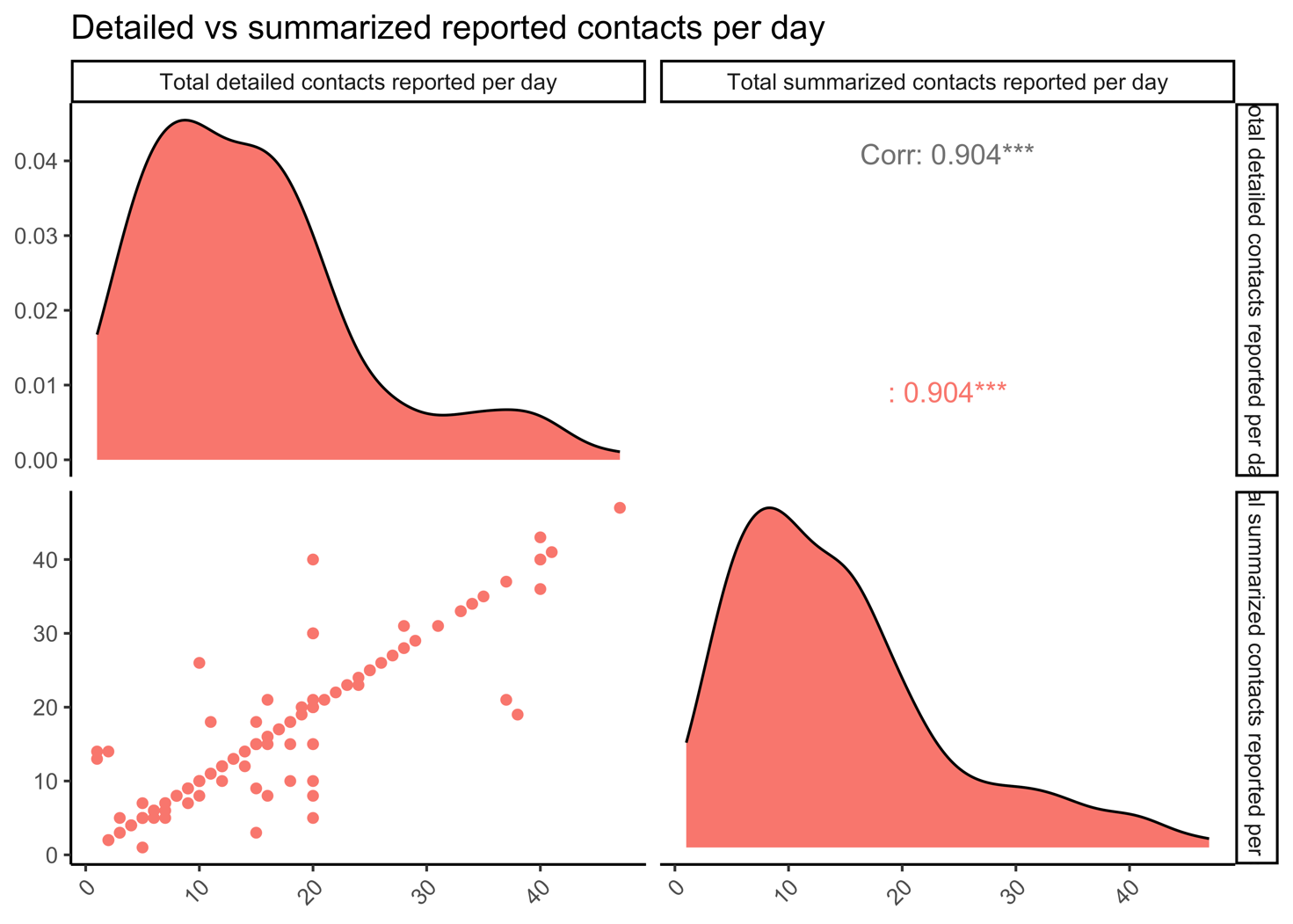


**Supplemental Bibliography**

1. Alsved M, Nyström K, Thuresson S, et al. Infectivity of exhaled SARS-CoV-2 aerosols is sufficient to transmit covid-19 within minutes. Sci Rep **2023**; 13(1): 21245.

2. Siegel JD, Rhinehart E, Jackson M, Chiarello L. 2007 Guideline for Isolation Precautions: Preventing Transmission of Infectious Agents in Health Care Settings. Am J Infect Control **2007**; 35(10 Suppl 2): S65-164.

3. Cattuto C, Van den Broeck W, Barrat A, Colizza V, Pinton JF, Vespignani A. Dynamics of person-to-person interactions from distributed RFID sensor networks. PLoS One **2010**; 5(7): e11596.

4. Leung NHL, Milton DK. New WHO proposed terminology for respiratory pathogen transmission. Nat Rev Microbiol **2024**; 22(8): 453-4.
